# Anatomical Organization of Urocortin 3-Synthesizing Neurons and Immunoreactive Terminals in the Central Nervous System of Non-Human Primates [*Sapajus* spp.]

**DOI:** 10.3389/fnana.2017.00057

**Published:** 2017-07-24

**Authors:** Daniella S. Battagello, Giovanne B. Diniz, Paulo L. Candido, Joelcimar M. da Silva, Amanda R. de Oliveira, Kelly R. Torres da Silva, Claudimara F. P. Lotfi, José A. de Oliveira, Luciane V. Sita, Cláudio A. Casatti, David A. Lovejoy, Jackson C. Bittencourt

**Affiliations:** ^1^Department of Anatomy, Institute of Biomedical Sciences, University of São Paulo São Paulo, Brazil; ^2^Center for Neuroscience and Behaviour, Institute of Psychology, University of São Paulo São Paulo, Brazil; ^3^Department of Anatomy, Santa Marcelina Medical School São Paulo, Brazil; ^4^Department of Basic Sciences, São Paulo State University, UNESP Araçatuba, São Paulo, Brazil; ^5^Laboratory of Cellular Structure and Function, Department of Anatomy, Institute of Biomedical Sciences, University of São Paulo São Paulo, Brazil; ^6^Institute of Biosciences, UNESP—São Paulo State University Botucatu, Brazil; ^7^Laboratory of Neuroendocrinology, Department of Cell and Systems Biology, University of Toronto Toronto, ON, Canada

**Keywords:** autonomic nervous system, corticotropin-releasing factor, monkey brain, paraventricular nucleus of the hypothalamus, corticotropin-releasing factor receptors, stress response

## Abstract

Urocortin 3 (UCN3) is a neuropeptide member of the corticotropin-releasing factor (CRF) peptide family that acts as a selective endogenous ligand for the CRF, subtype 2 (CRF_2_) receptor. Immunohistochemistry and *in situ* hybridization data from rodents revealed UCN3-containing neurons in discrete regions of the central nervous system (CNS), such as the medial preoptic nucleus, the rostral perifornical area (PFA), the medial nucleus of the amygdala and the superior paraolivary nucleus. UCN3-immunoreactive (UCN3-ir) terminals are distributed throughout regions that mostly overlap with regions of CRF_2_ messenger RNA (mRNA) expression. Currently, no similar mapping exists for non-human primates. To better understand the role of this neuropeptide, we aimed to study the UCN3 distribution in the brains of New World monkeys of the *Sapajus* genus. To this end, we analyzed the gene and peptide sequences in these animals and performed immunohistochemistry and *in situ* hybridization to identify UCN3 synthesis sites and to determine the distribution of UCN3-ir terminals. The sequencing of the *Sapajus* spp. UCN3-coding gene revealed 88% and 65% identity to the human and rat counterparts, respectively. Additionally, using a probe generated from monkey cDNA and an antiserum raised against human UCN3, we found that labeled cells are mainly located in the hypothalamic and limbic regions. UCN3-ir axons and terminals are primarily distributed in the ventromedial hypothalamic nucleus (VMH) and the lateral septal nucleus (LS). Our results demonstrate that UCN3-producing neurons in the CNS of monkeys are phylogenetically conserved compared to those of the rodent brain, that the distribution of fibers agrees with the distribution of CRF_2_ in other primates and that there is anatomical evidence for the participation of UCN3 in neuroendocrine control in primates.

## Introduction

Since the discovery of corticotropin-releasing factor (CRF) in the ovine hypophysis by Vale et al. (1981), several groups conducted studies in an attempt to find homologs sequences in other species. This culminated in the identification of other members that have structural and functional homology to CRF: Urocortin 1 (UCN1), discovered by Vaughan et al. (1995), Urocortin 2 (UCN2), described by Reyes et al. (2001) and Urocortin 3 (UCN3), reported by Lewis et al. (2001). In the present study, we will follow the suggested nomenclature for CRF receptors, CRF, subtype 1 (CRF_1_) and CRF, subtype 2 (CRF_2_), and their ligands, that was proposed by Hauger et al. (2003). UCN3, the newest member of the CRF family of peptides, is a 38-amino acid (aa) neuropeptide initially described and characterized in humans and rodents (Hsu and Hsueh, 2001; Lewis et al., 2001). This peptide was also described by Hsu and Hsueh (2001) and became known as Stresscopin (SCP). Analysis of residue or nucleotide sequences of UCN2 and UCN3 seems to indicate that these two neuropeptides are more related to each other than to other members of the “CRF family” (Lewis et al., 2001). Pharmacological studies demonstrate that both human and murine UCN3 bind to mouse CRF_2_ with high affinity but do not bind to murine CRF_1_ (Hsu and Hsueh, 2001; Lewis et al., 2001). This finding indicates a possible role of UCN3 as an endogenous ligand for the CRF_2_ receptor and that it has functional implications, such as the neuroendocrine control of food intake.

In rodents, messenger RNA (mRNA) encoding the UCN3 precursor molecule (ppUCN3) was detected in several peripheral organs and a few cortical and subcortical areas (e.g., the hypothalamus, amygdaloid complex and brainstem nuclei; Hsu and Hsueh, 2001; Lewis et al., 2001; Kang et al., 2007). This distribution is dissimilar to what has been reported for CRF (Swanson et al., 1983), UCN1 (Vaughan et al., 1995; Bittencourt et al., 1999) and UCN2 (Reyes et al., 2001). In the central nervous system (CNS) of rodents, UCN3 is found in the median preoptic nucleus (MnPO), the rostral perifornical area (PFA) that encompasses areas just lateral to the paraventricular nucleus (PVH), the posterior part of the bed nucleus of *stria terminalis* (BSTp) and the medial amygdaloid nucleus (MeA; Lewis et al., 2001).

The non-human primates *Sapajus libidinosus* and *Sapajus nigritus* (*Sapajus* spp.) are sympatric in some areas of South America and were previously designated *Cebus apella*. They belong to the New World monkeys, and they are among the Neotropical primate species that are commonly used for biomedical research (Silva, 2001; Fragaszy et al., 2004; Alfaro et al., 2012; Izar et al., 2012; Lynch Alfaro et al., 2012). Because the organization of the nervous system in these species is highly conserved across and within the vertebrate taxa, their brains are largely similar to other primates (Fragaszy et al., 2004).

Data regarding the distribution of UCN3 immunoreactive (UCN3-ir) cell bodies, fibers and terminals, and UCN3 mRNA expression are non-existent for primates. Also, *Sapajus* spp. is a suitable primate model for comparison with other mammalian model studies, such as those performed in rodents. Therefore, the goal of this study was to map UCN3-producing or ppUCN3 mRNA-expressing neurons in addition to UCN3-ir fibers and terminal fields in the *Sapajus* spp. monkey brain as a basis for beginning to understand the role of UCN3 in the CNS of primates.

## Materials and Methods

### Animals and Housing

Three young male tufted capuchin monkeys (*Sapajus* spp.), weighing 2–3 kg, were available for use in the present study and were group housed in a *vivarium* with water and food available *ad libitum*. The monkeys were provided by the Tufted Capuchin Monkey Procreation of the Araçatuba Dentistry School at São Paulo State University (UNESP) in Brazil and this study was carried out in accordance with the recommendations of the Brazilian Institute of Environment and Renewable Natural Resources (IBAMA) regulations and the Institute of Biomedical Sciences Ethical Committee for Animal Research guidelines (approved protocol #40/2009).

### Perfusion and Histological Preparations

The animals were anesthetized by intraperitoneal injection (ip) of sodium pentobarbital (30 mg/kg) and perfused transcardially (±1 liter/10 min) with 1 liter of 0.9% saline. This step was followed by 2 liters of cold 4% formaldehyde in 0.1 M sodium acetate buffer (pH 6.5) and 2 liters of cold 4% formaldehyde in 0.1 M sodium tetraborate fixative (pH 9.5). Next, the brain, spinal cord and hypophysis were dissected and cryoprotected in two solutions: (1) 10% glycerol and 2% DMSO with 0.1 M sodium tetraborate buffer (pH 9.5) for 3 days at 4°C; and (2) 20% glycerol and 2% DMSO in 0.1 M sodium tetraborate (pH 9.5) for 4 days at 4°C. We followed a formerly described protocol by Bittencourt et al. (1999), what requires no post-fixation time, which promoted superior staining in the immunohistochemical procedures. Next, frontal plane sections of neural axis were obtained in a freezing microtome (SM2000R, Leica, Wetzlar, HE, Germany) with 40 μm thickness in eight series and were stored in antifreeze solution at −30°C.

The hypophysis was processed for paraffin embedding and 5 μm thick histological sections were obtained in a rotary microtome (RM2155, Leica Microsystems, Wetzlar, HE, Germany) and collected the slices on positively charged glass slides (Knittel adhesive slides, Braunschweig, LS, Germany). The histological sections were dried at room temperature, kept at 37°C for 2 days in a laboratory incubator and then at 57°C for 1 h. The sections were deparaffinized in xylene and then dehydrated in descending grades of alcohol to distilled water. For cytoarchitectonic purposes, one series of slices from each animal undergone Nissl staining (0.025% thionin). We followed the parcellation described in “A Stereotaxic Atlas of the Brain of the Cebus Monkey” by Manocha et al. (1968), with additional data from “The Rhesus Monkey Brain—In stereotaxic coordinates” by Paxinos et al. (2009). Comparisons to New World monkeys were based on “The marmoset brain in stereotaxic coordinates” by Paxinos et al. (2012).

### UCN3 Cloning and Sequencing

#### Genomic DNA Extraction

The DNA extraction procedure was modified from Blin and Stafford (1976). Genomic DNA was utilized for the cloning of UCN3 because there are no intronic sequences in the prepro-hormone encoding gene. One gramme of frozen monkey liver was pulverized into a powder using a mortar and pestle pre-chilled with liquid nitrogen. The powdered tissue was incubated with extraction medium (10 mM Tris-HCl (pH 8.0), 0.1 M EDTA, 0.5% w/v sodium dodecyl sulfate, and 20 μg/ml DNAse-free pancreatic RNAse) at 37°C for 1 h in a shaking incubator. Proteinase K (100 μg/ml) was added to the lysate and incubated in a 50°C water bath for 150 min. The undigested material was isolated by centrifugation and 1 ml of the supernatant undergone phenol extraction which consisted in mixing samples with an equal volume of phenol and Tris-HCl solution pH 8.0 several times by inversion. The samples were then centrifuged twice at 5000 rpm for 10 min. The phenol fractions were discarded, the aqueous fraction was mixed with the same volume of chloroform-isoamyl alcohol twice, and then the chloroform fractions were discarded. Next, 2.5 volumes of cold 100% ethanol were added to 0.1 volume of 3 M sodium acetate and then mixed with the aqueous phase to obtain a DNA precipitate. The precipitated DNA was submitted to centrifugation 5000 rpm for 5 min, followed by two washes in 1 ml of 70% ethanol, and then it was dried briefly to allow the evaporation of ethanol. The pellet was mixed with 250 μl of water to dissolve the genomic DNA. The spectrophotometry was used to determine the DNA concentration, by using a 260/280 absorbance ratio (A_260/280_).

#### Polymerase Chain Reaction (PCR) Procedures

To determine the correct sequence that amplified the translated region of the monkey *UCN3 (mUCN3)* gene, two kits of oligonucleotides were used, which were based on the human *UCN3* sequence. Due to the elevated level of conserved sequence in the mammalian *UCN* genes, the primers were designed to hybridize with flanking untranslated regions of the *UCN3* gene. For the amplification reactions, the nest and semi-nested PCR methods were performed to reduce the non-specific binding, which consisted in a first amplification of the long DNA sequence by using specific primers and then the amplified DNA was used for a second amplification reaction. The PCR occurred in two reactions with a total volume of 50 μl (per reaction) containing 29 μl of sterile water (RNAse and DNA free), 5 μl of PCR buffer supplied with (NH_4_)_2_SO_4_ and 1.0 mM MgCl_2_ (Fermentas, Mississauga, ON, Canada), 2 μl of each 10 mM dNTP (400 μM in the final solution, R0192, Fermentas, Mississauga, ON, Canada), 120 ng of DNA, four units of Taq DNA polymerase (1 U/1 μl, EP0404, LC Recombinant; MBI-Fermentas) and 2 μl of each primer (0.2 μM in the final solution). Primer sequences were as follows: F1 (5′-atgctgatgccggtccacttc-3′) and R1 (5′-tcctccaatttgcgcatc-3′). The thermal cycling program consisted of in an initial denaturation phase for 3 min at 94°C, and for an amplification sequence of 35 1-min cycles at 94°C, 60°C, and 72°C. The program concluded with an extension phase at 72°C for 5 min. For the second PCR, 3 μl of F1/R1 were used as the template, the pairs R1 and F2 (5′-cgcaccaagttcaccctgtccctcga-3′) or R2 (5′- cctgctgagcaagaggagcttccac-3′) and F1 were carried out using the same method described previously. The PCR procedures were performed in an Eppendorf Mastercycler thermal cycler (Eppendorf Scientific Inc., Hamburg, Germany).

All the PCR-generated amplification products were separately ligated into the pCR^®^2.1-TOPO^®^ plasmid vector (K4550-40, TOPO TA cloning kit, Invitrogen, Carlsbad, CA, USA) and subcloned using TOP10 cells. Plasmid DNA sequences containing the cloned inserts were isolated using an Eppendorf Perfectprep plasmid miniprep kit (Eppendorf Scientific Inc., Hauppauge, NY, USA). The cloned fragment sizes were confirmed by restriction digestion with EcoR1. All cloned fragments were sequenced commercially (ATCG Corp., Toronto, ON, Canada). The subcloned sequences were analyzed by searching for nucleotide and protein sequence similarity using FASTA and NCBI BLAST + software (EMBL-EBI).

### *In Situ* Hybridization

To investigate and localize the *mUCN3* mRNA, we employed ^35^S-labeled antisense cRNA probes in the *in situ* hybridization protocol, which was adapted from Simmons et al. (1989). Full or partial series of brain sections from three monkeys were perfused and cut as previously described. The tissue was briefly mounted onto adhesion superfrost plus slides (Brain Research Lab, Newton, MA, USA) and fixed in 4% formaldehyde in 0.1 M phosphate buffered saline (PBS) for 5 min. Protein digestion was performed with 0.001% proteinase K (10 mg/ml) for 15 min at 37°C. The samples were then subject to the acetylation by 0.25% acetic anhydride in 0.1 M triethanolamine (TEA) pH 8.0 for 10 min, followed by rinses in 2× saline-sodium citrate (SSC). Finally, the tissue was dehydrated in ascending concentrations of ethanol and air dried.

Antisense *mUCN3* probes were produced from a 323-bp *mUCN3* cDNA which included the entire coding sequence (GenBank n° BC100870.1). The plasmid was designed by subcloning a PCR fragment encoding the part of the coding region of *mUCN3* into the EcoRI sites of pCR2.1-TOPO (K4550-40, TOPO TA cloning kit, Invitrogen, Carlsbad, CA, USA). Labeled antisense *mUCN3* probes were produced by linearizing the plasmid with XbaI enzyme. Next, the appropriate RNA polymerase (T7 for anti-sense) was used to synthesize the *mUCN3* probe (323 pb). Please, see Supplementary Figure S1 for probe details. The UCN3 probe fragment length was adjusted to 200 nucleotides to reduce alkaline hydrolysis, following the protocol established by Cox et al. (1984) and which was previously described for monkey tissue (Vasconcelos et al., 2003).

The probes were used at concentrations of approximately 10^6^ cpm/ml in a solution containing 50% formamide, 0.01% SDS, 0.3 M NaCl, 0.2% 5 M dithiothreitol, 10 mM Tris (pH 8.0), 0.01% tRNA, 1 mM EDTA pH 8.0, 1× Denhardt’s solution, and 10% dextran sulfate. Next, this hybridization solution (200 μl per section) was applied to monkey tissues and incubated at 56–58°C, overnight. Monkey sections were treated with 0.002% RNase A (20 μg/ml) in a solution containing 0.5 M NaCl and 1 mM EDTA (pH 8.0) at 37°C water bath for 30 min. After, the sections were submitted to gradually de-salt (stringency washes) with 1 mM DTT: 2× SSC at 50°C water bath for 1 h, 0.2× SSC at 55°C water bath for 1 h and, 0.2× SSC at 60°C water bath. Sections were dehydrated in a solution of 70% ethanol with 0.02% 2× SSC and 0.02% 5 M DTT for 10 min, air dried at room temperature. The sections were dipped in Kodak NTB-2 liquid autoradiographic emulsion, dried at 37°C for 3 h and then exposed for an average of 4 weeks at 4°C. The slides were developed with Kodak D-19 and counterstained with 0.25% thionin for reference purposes.

### Antisera Characterization

All antisera used in this study have been characterized in other studies, and they specifically label the expected group of cells (Li et al., 2002; Chen et al., 2011). Labeling specificity was also confirmed by omitting the primary antibodies and by using negative and positive controls in serum with 5 mg/ml heparin and 2% bovine serum albumin (blocking solution). To ensure optimal staining with the antisera, we titrated the antibodies as described by Hoffman et al. (2008; see Supplementary Figure S2 for titration details). Please refer to Table 1 for additional information regarding the antisera used in the present study.

**Table 1:** Primary antibodies used in the experiments.

Common name of antibody	Manufacturer	Number or code	References	PubMed ID	Antibody ID from NIF	Antibody link	Amino acid position	Dilution
Rabbit anti-human urocortin 3 serum [anti-hUCN3]	Peptide Biology Laboratory, Dr. Joan Vaughan	PBL# 6570	Li et al. (2002)	11826127	AB_2315526	http://antibodyregistry.org/AB_2315526	-	1:7000 [peroxidase]
								1:2000
								1:7000 [fluorescence]
Rabbit anti-human urocortin 3 serum [anti-hUCN3]	Peptide Biology Laboratory, Dr. Joan Vaughan	PBL# 7218	van der Meulen et al. (2015)	26076035	-	-	-	1:7000 (peroxidase)
								
								
Rabbit anti-rat/human corticotropin-releasing factor serum (anti-r/hCRF)	Peptide Biology Laboratory, Dr. Joan Vaughan	PBL# rC70	Sawchenko et al. (1984)	6609226	-	http://antibodyregistry.org/AB_2314234	-	1:3000 (fluorescence)

To ensure the specificity of the antibodies, we used two sets of controls. In the first set, the same immunohistochemistry procedure used to identify UCN3-ir in monkey brain was employed in tissue from both WT mice (wild-type UCN3, *wt/UCN3*) and mice that had the *UCN3* gene deleted (“knockout” UCN3, *ko/UCN3)*. These mice were kindly provided by Dr Kuo-Fen Lee from the Peptide Biology Laboratory at The Salk Institute for Biological Studies in La Jolla, CA, USA. The second control employed was a competitive adsorption test, where the antisera were preincubated (overnight at 4°C) with 0–250 μM synthetic UCN3 (hUCN3, #415-174-15) or related peptides belonging to the CRF family, such as CRF itself (r/hCRF, #365-198-15) and UCN1 (rUCN, #351-156-15) and the staining patterns and intensities were analyzed. Peptides were generously provided by Dr Jean Rivier of the Laboratories for Peptide Biology at the Salk Institute. Please, see Supplementary Figures S3–S5 for more details.

### Immunohistochemistry

The “free-floating” sections were briefly pre-treated with a solution of 0.3% hydrogen peroxide (H_2_O_2_) diluted in 0.02 M potassium phosphate buffer (KPBS, pH 7.4). The tissue was rinsed in KPBS and then was incubated for 48 h at 4°C in the primary anti-hUCN3 antiserum at 1:7000 (see Table 1 for antisera details) with blocking solution (described below). After incubation in primary antibody, the monkey brain sections were washed in KPBS and incubated in a biotin-conjugated secondary antibody solution for 1 h (1:200; Jackson Immunoresearch Laboratories, West Grove, PA, USA), rinsed in KPBS and then incubated with an avidin-biotin complex solution for 1 h (1:100, Vectastain Elite; Vector, Burlingame, CA, USA). After KPBS rinses, the tissues were submitted to the peroxidase reaction using H_2_O_2_, 3,3′-diaminobenzidine (DAB, Sigma-Aldrich, St. Louis, MO, USA) as chromogen and nickel ammonium sulfate (NAS, Fisher Scientific, Pittsburgh, PA, USA) as a reaction amplifier. Standard procedures were followed. However, some previously described modifications that had been shown to improve UCN3-ir cell and fiber labeling (Bittencourt et al., 1999) were performed: (1) not pre-treating tissue with sodium borohydride; and (2) incubating tissue in a blocking solution containing 5 mg/ml of heparin, 2% bovine serum albumin, 3% Triton X-100 in KPBS solution for all incubations.

Besides, we used the indirect immunofluorescence technique to study the localization of UCN3 and CRF in the paraventricular nucleus of the hypothalamus (Pa). For UCN3 localization, sections from three different animals were rinsed in KPBS and were incubated for 1 h in KPBS solution containing 0.03% Triton X-100, 1% bovine serum albumin, 5 mg/ml heparin (blocking solution), followed by incubation over 48 h at 4°C in anti-hUCN 3 antiserum (1:2000, see Table 1 for details of the antisera) diluted in the blocking solution. After rinses in KPBS, sections were incubated in Alexa Fluor^®^ 594 donkey anti-rabbit secondary antibody (1:200, Thermo Fisher Scientific, Waltham, MA, USA) diluted in the same blocking solution, for 2 h at room temperature. After KPBS washes, sections were mounted onto gelatin-coated slides and coverslipped with buffered glycerol medium. For CRF localization, adjacent sections from the same three different animals previously described were rinsed in KPBS, were incubated in blocking solution for 1 h, and then were incubated in anti-r/hCRF (rat/human CRF) antiserum (1:3000, in the same blocking solution; see Table 1 for antisera details) over 48 h at 4°C. Next, the sections were incubated in Alexa Fluor^®^ 488 donkey anti-rabbit secondary antibody (1:200, Thermo Fisher Scientific, Waltham, MA, USA) diluted in the same blocking solution, for 2 h at room temperature. After KPBS washes, sections were mounted onto gelatin-coated slides and coverslipped with buffered glycerol medium.

We also employed the indirect immunofluorescence technique to study the localization of UCN3 in the hypophysis. Briefly, the sections were washed in 0.1 M PBS, pH 7.4 and subjected to antigen retrieval using sodium citrate buffer (10 mM sodium citrate, 0.05% Tween 20, pH 6.0, Sigma-Aldrich, St. Louis, MO, USA) under heat and humid pressure (Decloaking chamber, Model DC2002, Biocare Medical, Pacheco, CA, USA) at 95°C for 5 min. The sections were cooled to room temperature and subjected to non-specific blocking using 5% non-fat milk, followed by secondary blocking using 3% BSA in PBS/0.3% Triton X-100 and incubated with anti-hUCN3 antiserum (1:7000, see Table 1 for antisera details) at 4°C for 48 h. The sections were first incubated with the specific secondary antibody, followed by streptavidin conjugated with Cy3 (1:500, Jackson Immunoresearch, West Grove, PA, USA), and then they were counterstained with DAPI nuclear stain (Biosensis, Thebarton, SA, Australia). Finally, the histological sections were preserved with buffered glycerol mounting medium and coverslips.

### Imaging and Data Analysis

The images were acquired with a Digital Sight DS-Ri1 digital camera (Nikon Corporation, Tokyo, Japan) coupled to a Leica microscope (Leica, Wetzlar, HE, Germany) using the image capture software NIS—Elements BR 3.14 (Nikon Corporation, Tokyo, Japan). For the analysis of UCN3 immunofluorescence, the slides were examined under a Leica EL6000 microscope equipped with epifluorescence. The digital photomontages were obtained with a Nikon ECLIPSE 80i microscope connected to a Dell computer that was fitted with the Neurolucida Suite (MicroBrightField, Inc.—MBF, Williston, VT, USA).

Single UCN3-ir and CRF-ir cells in the Pa were analyzed using a confocal laser scanning microscope Zeiss LSM 780 NLO (CarlZeiss AG, Jena, TH, Germany) located at the Centro de Facilidades de Apoio à Pesquisa (CEFAP/USP) at the University of São Paulo.

**Figure 1:**
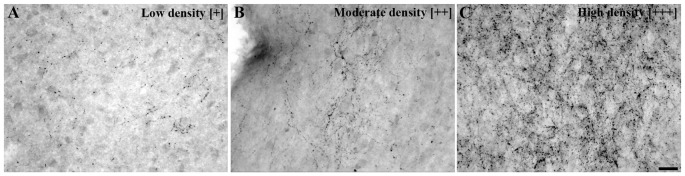
Urocortin 3 immunoreactivity (UCN3-ir) fiber density scale. Bright field photomicrographs showing various UCN3-ir fiber and terminal projection densities: **(A)** low density [+], **(B)** moderate density [++], **(C)** high density [+++] and [−] indicating a complete absence of specific labeling. This scale was used as a reference to perform the fiber density evaluation presented in Table 3. Scale bar: 25 μm **(A–C)**.

The presence of cells containing *UCN3* mRNA and UCN3-ir cell bodies and fibers were individually evaluated through semiquantitative analysis among the positively labeled regions according to a previously defined rating scale (Figure 1). All images were cropped and adjusted for color balance, brightness, sharpness, and contrast using Adobe Photoshop CS 5.1 version 12.1, 2011.

## Results

### Gene Sequencing of *Sapajus* spp. *UCN3*

**Figure 2:**
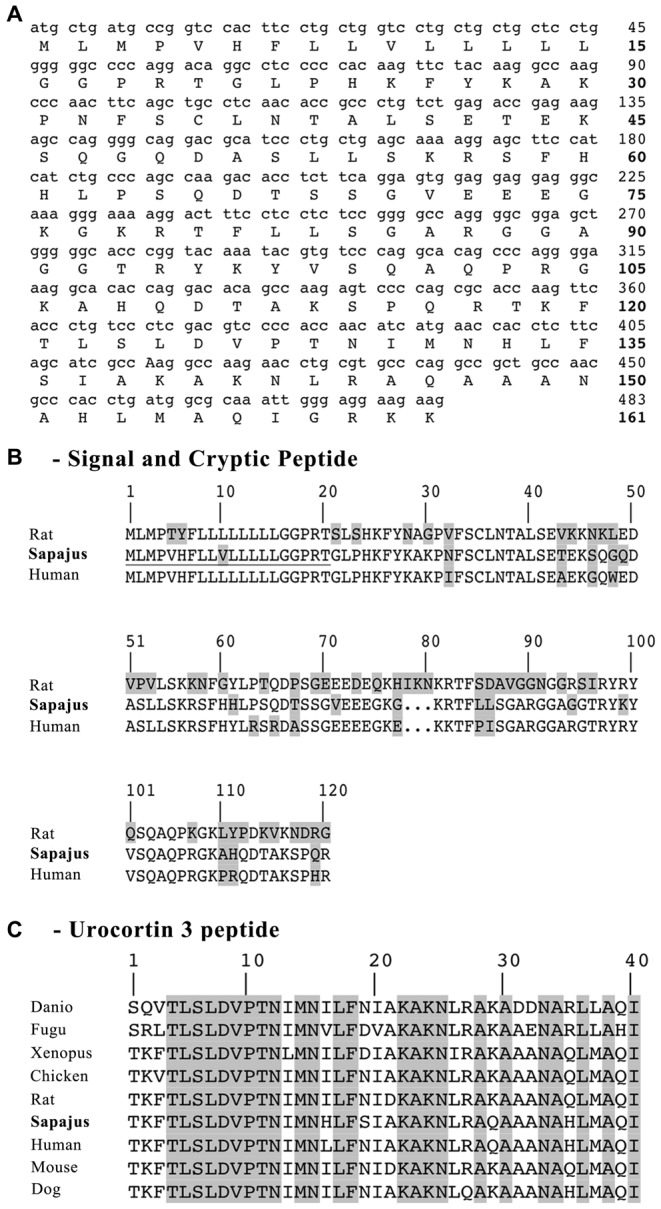
*Sapajus* prepro-UCN3 (ppUCN3) structure. **(A)** Nucleotide and amino acid (aa) sequences of the *Sapajus* prepro-hormone. Corresponding aa are shown below each codon. The numbers on the right indicate base position (normal text) or residue position (boldface). **(B)** Comparison of the primary structures of rat, monkey and human prepro-UCN3. Boxed residues indicate mismatches relative to the other two sequences. Sequences have been aligned for best fit. **(C)** Comparison of UCN3 residue sequences of Danio, *Danio rerio*; Fugu, *Tetraodon nigroviridis*; Xenopus, *Xenopus laevis* and *tropicalis*; Chicken, *Gallus*; Rat, *Rattus norvegicus*; Sapajus, *Sapajus* spp.; Human, *Homo sapiens*, Mouse, *Mus musculus* and Dog, *Canis lupus*. Boxed regions indicate residues that are shared among species.

The *mUCN3* gene is 483 bases in length (Figure 2) and has 88% sequence identity relative to its human cDNA sequence correspondent to the matched region. At the aa level, the *Sapajus* sequence has 120 aa, of which 21 residues (17.5%) are substitutions relative to human ppUCN3 and 61 residues are substitutions (50.83%) relative to rat ppUCN3 (Figure 2). As in humans, the monkey ppUCN3 aa sequence has a three-residue deletion compared to the rat sequence at positions 78–80. The supposed mature sequence of mUCN3 is also 40 aa in size (Figure 2), and only two and four substitutions are related to the human and rat sequences, respectively. No one of the modifications in the mature UCN3 monkey sequence is feasible to preclude functional differences in the peptide.

### Antisera Specificity

**Figure 3:**
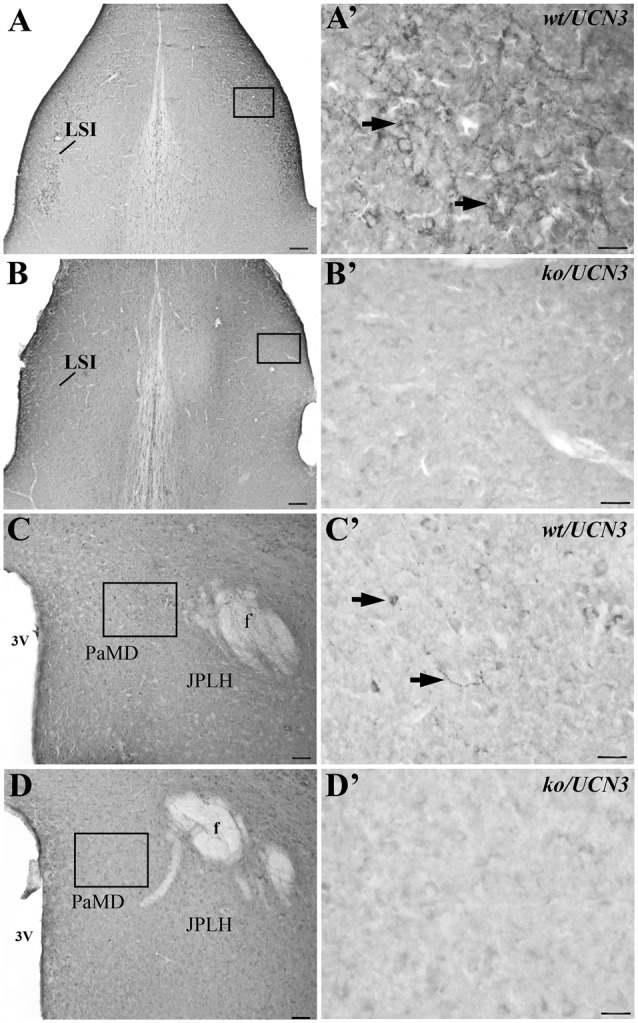
Representative photomicrographs illustrating UCN3 immunostaining in the intermediate part of the lateral septal nucleus (LSI) and paraventricular hypothalamic nucleus, magnocellularpart, dorsal division (PaMD) regions of wild-type UCN3 (*wt/UCN3*) mice or “knockout” UCN3 (*ko/UCN3*) mice. **(A)** Bright field photomicrograph showing the LSI region of a *wt/UCN3* mouse. **(A′)** Higher magnification of **(A)** UCN3-ir cells and fibers, indicated by black arrows. **(B)** Bright field photomicrograph showing the LSI region of a *ko/UCN3* mouse. **(B′)** Higher magnification of the square area in **(B)**. Note the absence of labeling. **(C)** Bright field photomicrograph revealing the PaMD region of a *wt/UCN3* mouse. **(C′)** Higher magnification of the square area in **(C)** showing UCN3-ir cells and fibers, indicated by black arrows. **(D)** Bright field photomicrograph the PaMD region of a *ko/UCN3* mouse. **(D′)** Higher magnification of the square area in **(D)**. Note the absence of labeling. Scale bars: 100 μm **(A,B)**; 50 μm **(C,D)**; 20 μm **(A′–D′)**.

Both antisera (PBL#6570 and PBL#7218) proved to be specific, based on the results of the controls studies performed. Table 2 contains the results of the competition studies. All UCN3-ir staining in the Pa and in the ventromedial nucleus of the hypothalamus (VMH), which were detected in the control group, were abolished by pre-incubation of the UCN3 antibody for the total UCN3 peptide concentrations employed. Pre-adsorption with CRF or UCN1 diminished but did not eliminate, staining of UCN3-ir cells in the Pa or fibers in the VMH when compared to the positive control group. Furthermore, while in *wt/UCN3* mice it was possible to observe UCN3-ir cells and fibers in regions that had previously described UCN3-ir (Li et al., 2002), all reactions in *ko/UCN3* mice returned negative results (Figure 3).

**Table 2:** Competition studies of Urocortin 3 immunoreactivity (UCN3-ir) in the *Sapajus* spp. monkey brain.

Competing peptide	Staining	
[concentration in mg/ml]	Pa (UCN3-ir cells)	VMHDM (UCN3-ir fibers)
**UCN3**		
Control	+	++++
1 mg/ml	−	−
0.5 mg/ml	−	−
0.1 mg/ml	−	−
0.05 mg/ml	−	−
0.01 mg/ml	−	−
**UCN1**		
Control	+	+++
1 mg/ml	+	+++
0.5 mg/ml	+	+++
0.1 mg/ml	+	+++
0.05 mg/ml	+	+++
0.01 mg/ml	+	+++
**CRF**		
Control	+	+++
1 mg/ml	+	+++
0.5 mg/ml	+	+++
0.1 mg/ml	+	+++
0.05 mg/ml	+	+++
0.01 mg/ml	+	+++

*Staining intensity was rated using a four-point scale: [++++] denotes when labeling intensity was indistinguishable from that achieved using the control antisera [non-pre-adsorbed] and [−] indicates a complete absence of specific labeling. The number of plus signs for the ventromedial nucleus of the hypothalamus, dorsomedial part (VMHDM) represents the density of UCN3-ir fibers, and for paraventricular nucleus of the hypothalamus (Pa), [+] accounts for presence of UCN3-ir cells and [−] represents their absence*.

### Cellular Localization of UCN3 Immunoreactivity and mRNA

**Figure 4:**
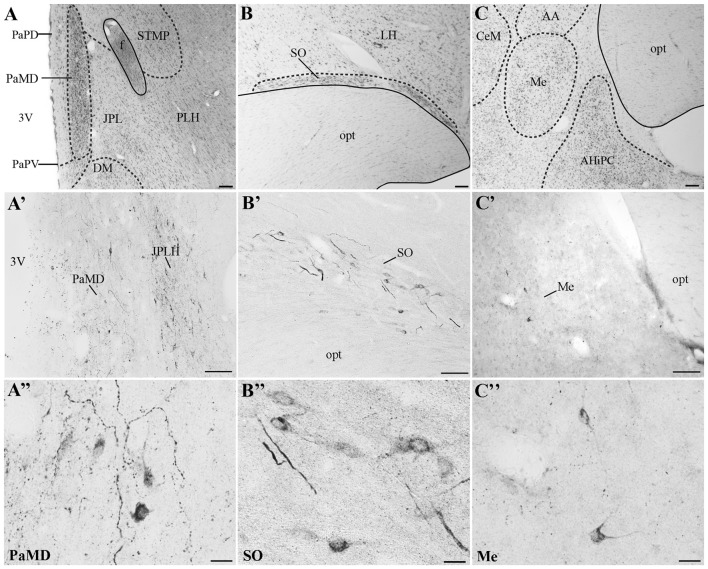
Distribution of UCN3-ir cells in the central nervous system (CNS) of *Sapajus* spp. A series of bright field images showing representative regions that display the highest densities of UCN3-ir labeled cells. Nissl stained regions of **(A)** hypothalamus, **(B)** supraoptic nucleus, **(C)** amygdala. Dashed lines show nuclear boundaries. **(A′,A″)** Bright field photomicrographs demonstrating UCN3-ir neurons in the PaMD and juxtaparaventricular part of lateral hypothalamus (JPLH; **B′,B″**) in the SO and, **(C′,C″**) in the Me. Scale bars: **(A,C)** 200 μm; **(B,A′,B′,C′**) 100 μm; **(A″–C″)** 20 μm.

In all monkeys examined, we observed UCN3-ir cells were restricted to a few regions in the CNS, mainly in the hypothalamic and amygdaloid regions (Figure 4; Table 3). In the hypothalamus, UCN3-ir neurons were located primarily in the juxtaparaventricular part of the lateral hypothalamus (JPLH), which was located medial to the fornix (f; Figure 4A). This nucleus, in a rostral position in the forebrain, showed the most intense immunostaining when the JPLH was located medial to the fornix and ventrolateral to the dorsal magnocellular part of the Pa (PaMD) and the dorsal parvocellular part of the Pa (PaPD; Figures 4A′,A″). Neuronal bodies were also found in the periventricular hypothalamic nucleus (Pe), to which the full extent bordered the 3rd ventricle (3V). Few UCN3-ir cells were detected in the supraoptic nucleus (SO), dorsal to the optic tract (opt), dorsolateral to the supraoptic decussation (sox), ventral to the lateral hypothalamic area (LH) and lateral to the anterior hypothalamic area (AH; Figures 4B–B″). There were no UCN3-ir cells in the preoptic area or the MnPO.

**Table 3:** Distribution of UCN3-ir neurons and fibers and *UCN3* messenger RNA (mRNA) in *Sapajus* spp.

Cell group	UCN3-ir neurons	UCN3-ir fibers	*UCN3* mRNA
Forebrain			
Hippocampal formation			
Dentate gyrus of hippocampal			
formation	−	−	+
Field CA1	−	−	+
Amygdala			
Medial nucleus	+	+	−
Centromedial nucleus	−	++	+
Septal region			
Medial septal nucleus	−	+	−
Lateral septal, intermediate part	−	+++	−
Bed nucleus of stria terminalis			
Anterior part	−	+	+
Ventral part	−	+	+
Diencephalon			
Thalamus			
Paraventricular nucleus	−	+	−
Medial habenula	−	+	−
Hypothalamus			
Periventricular zone			
Median preoptic nucleus	−	−	+
Anteromedial preoptic nucleus	−	−	+
Supraoptic nucleus	+	+	+
Paraventricular nucleus			
Dorsal magnocellular part	+++	+++	+
Dorsal parvocellular part	+	+	+
Ventral parvocellular part	+	+	−
Juxtaparaventricular lateral nucleus	+++	+++	+
Periventricular nucleus	+	++	−
Median eminence, external zone	−	+	−
Arcuate nucleus	+	++	−
Medial zone			
Lateral preoptic nucleus	−	++	+
Anterior hypothalamic nucleus	−	−	+
Ventromedial nucleus of			
hypothalamus	−	+++	−
Lateral zone			
Lateral hypothalamic area	−	+	−
Brainstem			
Periaqueductal gray, lateral part	−	+	−
Periaqueductal gray,			
ventrolateral part	−	+	−
Spinal trigeminal tract nucleus,			
caudal part	−	+	−
Nucleus of solitary tract	−	+	−
Dorsal motor nucleus of vagus,		
centrointermediate part	−	+	−
Hypophysis			
anterior lobe	−	−	−
posterior lobe	−	++	−
intermediate lobe	−	−	−

*The presence of UCN3-ir neurons and fibers, as well as *UCN3* mRNA, are indicated by a plus sign, and their absence is indicated by a minus sign. The relative density of UCN3-ir fibers in different brain regions agrees to the patterns shown in Figure 1, indicated by the number of plus signs depending on the amount present in a specific region or with a minus sign to indicate the absence of labeled fibers*.

In extrahypothalamic areas, UCN3-ir cells were predominantly found in amygdaloid areas, specifically in the medial amygdaloid nucleus (Me; Figures 4C–C″). A few UCN3-ir neurons were observed in the cortical nucleus of the amygdala (Co). There were no UCN3-ir cells in regions of the neocortex, brainstem or spinal cord.

**Figure 5:**
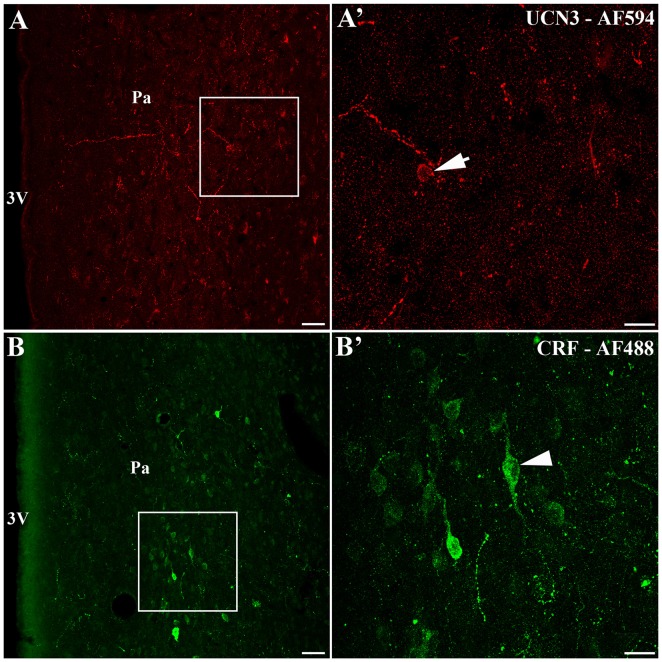
Localization of UCN3-ir and corticotropin-releasing factor (CRF)-ir labeled cells in the paraventricular nucleus of the hypothalamus (Pa) of *Sapajus* spp. **(A)** Confocal photomicrograph showing UCN3-ir cells located in the *Sapajus* Pa. **(A′)** Higher magnification of the square area in **(A)** demonstrating UCN3-ir labeled cell, indicated by the white arrow. **(B)** Confocal photomicrograph revealing CRF-ir cells in the region of Pa. **(B′)** Higher magnification of the square area in **(B)** showing CRF-ir labeled cells, indicated by the white arrowhead. Note the morphological difference between UCN3 (small neuron) and CRF (large neuron) labeled cells in the monkey Pa. Scale bars: **(A,B)** 50 μm; **(A′,B′)** 20 μm.

We identified the presence of UCN3-ir labeled cells located closely to the area that CRF-ir labeled cells are found in the Pa (Figure 5). Using confocal microscopy, we observed small UCN3-ir neurons mainly in the dorsal part of PaMD (Figures 5A,A′). In contrast, we detected large CRF-ir labeled neurons located primarily in the ventral part of the PaMD (Figures 5B,B′).

**Figure 6:**
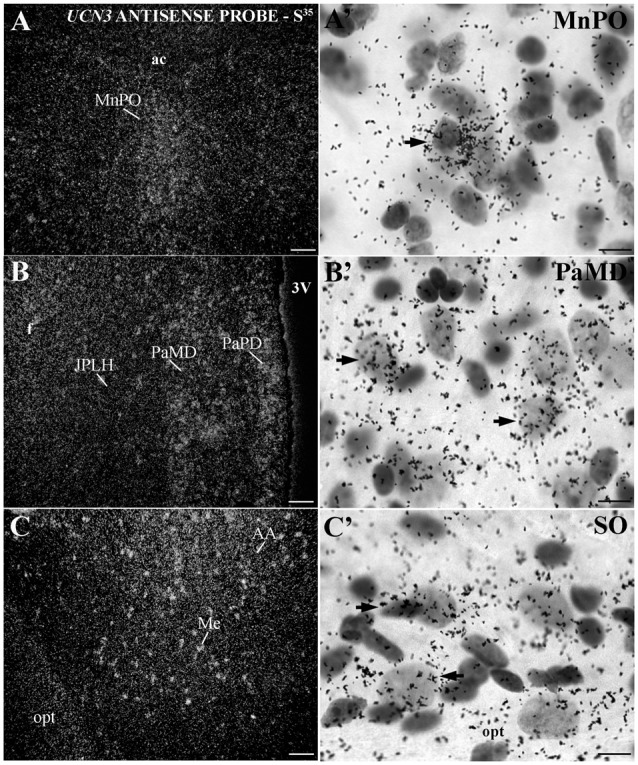
Distribution of *UCN3* messenger RNA (mRNA) in the CNS of *Sapajus* spp. **(A–C)** A series of darkfield photomicrographs showing *UCN3* mRNA expression (silver grains) in the median preoptic nucleus (MnPO), JPLH, PaMD, paraventricular hypothalamic nucleus,parvicellular part, dorsal division (PaPD) and medial amygdaloid nucleus (Me) regions; **(A′–C′)** bright field photomicrographs showing silver grains over Nissl-counterstained neurons at higher magnification. Scale bars: **(A–C)** 50 μm; **(A′–C′)** 10 μm.

Similar to the immunohistochemical method, the *in situ* hybridization technique allowed the visualization of *UCN3* mRNA expression in restricted regions of the monkey CNS, located predominantly in the hypothalamic and amygdaloid regions (Figure 6). Using light microscopy, we detected neurons with silver grains in the cytoplasm, indicative of *UCN3* mRNA expression, in the MnPO (Figures 6A,A′), PaMD and PaPD (Figures 6B,B′) and SO (Figures 6C,C′).

**Figure 7:**
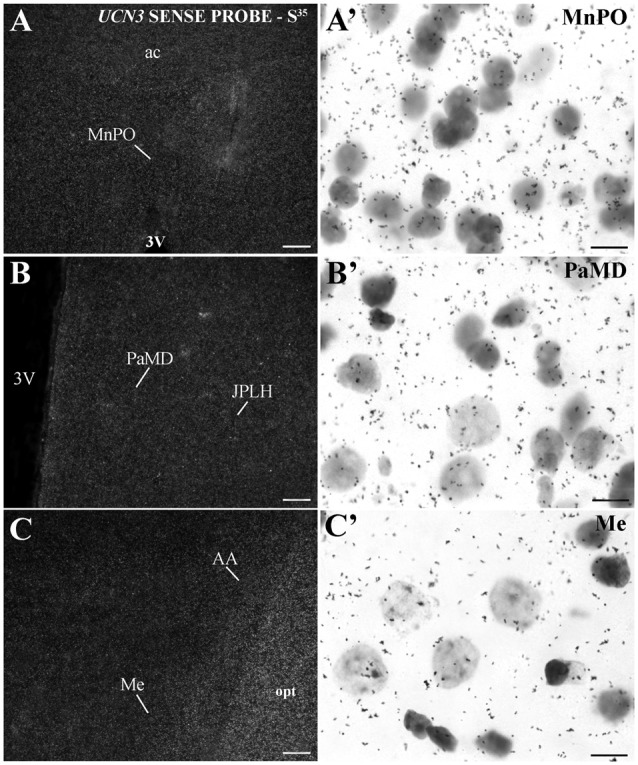
*UCN3* mRNA hybridization control in the CNS of *Sapajus* spp. A series of darkfield and bright field photomicrographs showing that when using a cRNA sense probe for prepro-UCN3, no labeled cells were hybridized. Scale bars: **(A–C)** 50 μm; **(A′–C′)** 10 μm.

In extrahypothalamic areas, cell bodies with *UCN3* mRNA expression were located in the following amygdaloid nuclei: CeM, basomedial amygdaloid nucleus (BM), Me and anterior amygdaloid area (AA) (Figure 6C). *UCN3* mRNA expression was also detected in other regions of the monkey CNS, such as the central medial thalamic nucleus (CM), centrolateral thalamic nucleus (CL), paracentral thalamic nucleus (PC), granule cell layer of the dentate gyrus (GrDG) and the anterior (STMA) and ventral (STMV) regions of the medial division of the bed nucleus of *stria terminalis* (ST). There was no detectable *UCN3* mRNA expression using the *UCN3* cRNA sense probe (Figure 7).

### UCN 3-ir Projections in the Monkey CNS

**Figure 8:**
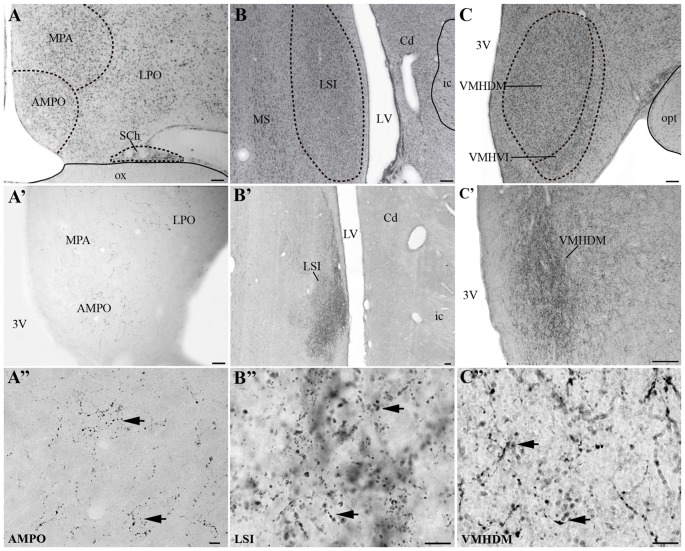
Distribution of UCN3-ir fibers in the CNS of *Sapajus* spp. A series of bright field photomicrographs of selective innervation by UCN3-ir fibers in discrete regions of the monkey brain. **(A–C)** Are Nissl-stained sections of representative areas with overlays indicating the structure boundaries.** (A′)** Preoptic area; **(B′)** LSI **(C′)** ventromedial nucleus of the hypothalamus, dorsomedial part (VHMDM). **(A″–C″)** Are higher magnification photomicrographs of **(A′–C′)** showing UCN3-ir fibers and terminals. Please note UCN3-ir fibers and varicosities (black arrows) and the very dense amount of UCN3-ir fibers in LSI and VMHDM in **(B′,C′)**. Scale bars: **(A–C)** 200 μm;** (A′)** 50 μm;** (B′,C′)** 100 μm; **(A″–C″)** 10 μm.

Visualization of fibers and terminal fields occurred predominantly in hypothalamic and limbic regions. The main sites of UCN3-ir fibers were the septal nuclei, such as the intermediate (LSI) and ventral parts (LSV) of the LS. The LSI was the main site of intense UCN3-ir (Figures 8B–B″). In the hypothalamus, the dorsomedial part of VMH (VMHDM) exhibited intense fiber and varicose UCN3-ir unlike the ventrolateral part of the VMH (VMHVL; Figure 8C). The most intense immunostaining for UCN3 was observed at the level where the VMHDM was located ventromedially to the caudal part of the fornix at the level of the lateral tuberal nucleus (LTN; Figures 8C′,C″).

We found moderate to high densities of UCN3-ir fibers in the JPLH, and this was associated with the presence of labeled neurons in this area. A considerable amount of UCN3-ir fibers and varicosities was observed in the following divisions of the Pa: PaMD, PaPD and the ventral division of the parvicellular part of the Pa (PaPV). We also found UCN3-ir fibers and varicosities in the preoptic hypothalamus, specifically in the anteromedial preoptic nucleus (AMPO), medial preoptic area (MPA) and lateral preoptic area (LPO; Figures 8A′,A″). In the AMPO, UCN3-ir fibers were mainly located in regions where this nucleus was located ventromedial to the MPA. The MPA was more innervated by UCN3-ir fibers when it was located more dorsolateral to the 3V, dorsomedial to the AMPO and medial to the LPO. In the LPO, fibers were present when it was located more lateral to the MPA and dorsolateral to the AMPO.

**Figure 9:**
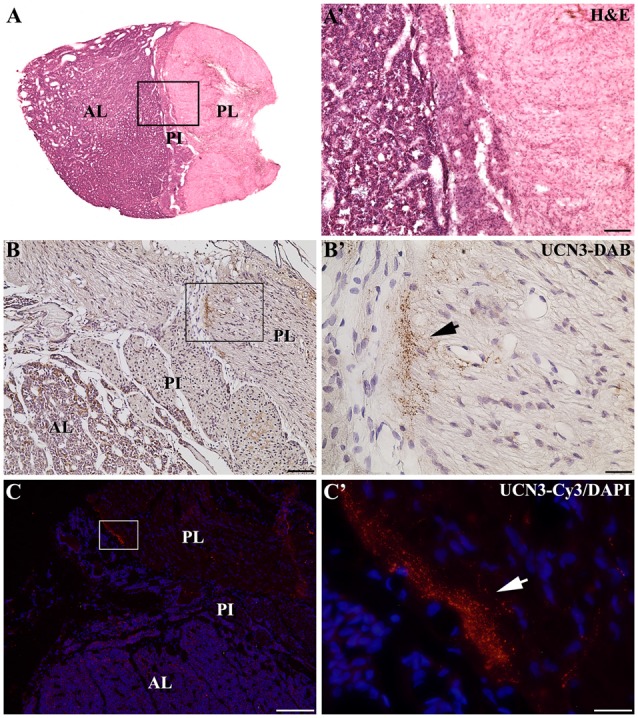
Distribution of UCN3-ir fibers in the hypophysis of *Sapajus* spp. **(A)** Photomontage of bright field photomicrographs of the entire *Sapajus* pituitary histology by H&E staining showing the clear distinction between the anterior lobe (AL), pars intermedia (PI) and posterior lobe (PL). **(A′)** Higher magnification of **(A)** demonstrating the boundaries between the AL, PI and PL. **(B)** Bright field photomicrograph showing UCN3-ir fibers in the posterior lobe.** (B′)** Higher magnification of the square area in **(B)** showing an UCN3-ir bundle of fibers, indicated by the black arrow; **(C)** Fluorescence photomicrograph of UCN3-ir fibers in the posterior lobe; **(C′)** higher magnification of the square area in **(C)** showing an UCN3-ir bundle of fibers, indicated by the white arrow. Scale bars: **(A′,B)** 100 μm; **(B′,C′)** 25 μm; **(C)** 200 μm.

**Figure 10:**
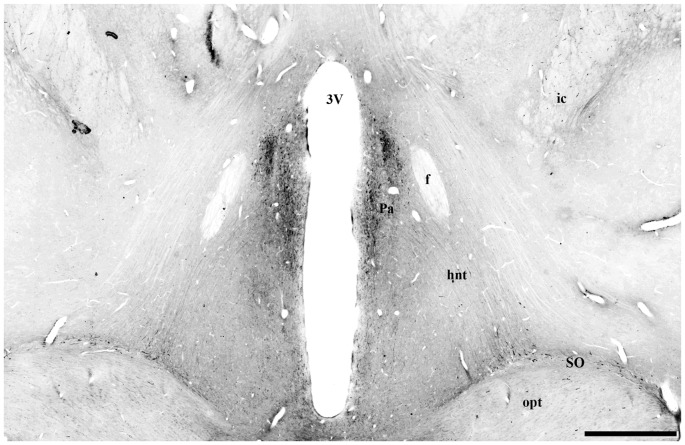
Digital photomontage of the hypothalamo-neurohypophysial tract (hnt) of *Sapajus* spp. Note the UCN3-ir cells in the paraventricular nucleus of the hypothalamus and their stained UCN3 fibers leaving this group of cells in a descending pathway towards the median eminence (ME) and posterior hypophysis through the hypothalamic-pituitary tract, which is visualized bilaterally. Scale bar: 1000 μm.

In the median eminence (ME), UCN3-ir fibers were observed in moderate intensity in the external layer (EL), but also in the arcuate hypothalamic nucleus (Arc), where these fibers were transiting to the ME, a pattern that is more evident at rostral levels. Moderately dense UCN3-ir fibers were restricted to the posterior lobe of the hypophysis (PL; Figure 9). Furthermore, it was possible to verify bilateral fibers of the monkey hypothalamo-neurohypophysial tract (hnt; Figure 10).

In extrahypothalamic areas, we found low to moderate UCN3-ir fiber densities in the Me, where it is located lateral to the *opt*, dorsal to the AA, dorsomedial to the CeM, and ventral to the basomedial amygdaloid nucleus, magnocellular part (BMMC) and the amygdalohippocampal area, parvicellular part (AHiPC). The STMA contains moderately dense UCN3-ir fibers. Few UCN3-ir fibers and varicosities were observed in the medial habenular nucleus (MHb).

In the mesencephalon, some fibers were found in the lateral periaqueductal gray (LPAG) and the ventrolateral periaqueductal gray (VLPAG). In the brainstem, there were few fibers and terminal fields in the solitary nucleus (Sol) and the caudal part of the spinal trigeminal nucleus (Sp5C). Terminal fields were also found in the centrointermediate part of the dorsal motor nucleus of vagus (CeI). There were no UCN3-ir fibers in the entire rostrocaudal axis of the spinal cord.

## Discussion

### Methodological Considerations

Two UCN3 antisera were employed in this study. The first antiserum, PBL#6570, was used for all experiments, including cell and fiber mapping. The second antiserum, PBL#7218, was utilized for the image acquisition in some immunohistochemical experiments, but it displayed less specificity when compared to PBL#6570 and was not used for mapping. Although PBL#6570 is not species-specific, the adsorption tests demonstrated that it recognized the antigen with high specificity. Additionally, this antiserum labeled the UCN3-ir in the Pa of brains of other mammals (Li et al., 2002; Chen et al., 2011). Both antisera failed to stain tissue from *ko/UCN3* mice, further demonstrating their specificity.

It is worth mentioning that we followed the parcellation for the Cebus monkey (Manocha et al., 1968), compared data to the common New World monkey (*Callithrix jacchus*, Paxinos et al., 2012) and adopted the nomenclature from the Rhesus monkey (Paxinos et al., 2009) to support our neuroanatomical analysis. This decision was taken because the *Sapajus* spp. (previously described as *Cebus apella*) is more comparable in size, sulcal pattern and habits to Old World monkeys of the *Macaca* genus than to New World monkeys (Gattass et al., 1987; Pinato et al., 2009). Albeit these differences complicate the interpretation of our results obtained using a New World Monkey atlas, we still compared the morphological aspects of our data across multiple species (rodents, Rhesus and Marmoset) to highlight conserved aspects of the UCN3 system.

Pre-adsorption with the UCN3 peptide eliminated labeling in both the Pa and VMH, what did not occur when CRF and UCN1 were used, although pre-adsorption with them diminished labeling. Indeed, among CRF-related peptides, hUCN3 was 32% similar to hCRF, 21% similar to hUCN1, 40% similar to mouse UCN2 and 37% similar to the human urocortin-related peptide (hURP; Lewis et al., 2001). Therefore, it is likely that those peptides could have some antigenicity to the antisera used in this study. The prepro-hormone sequence of these peptides differs considerably from that of the monkey or human ppUCN3, so it is unlikely that the cRNA probe would hybridize to UCN1, UCN2 or CRF prepro-mRNA. The *UCN3* mRNA in the CNS of monkey was mapped using a probe generated from the monkey cDNA sequence. Control studies with the sense probe resulted in no signal.

### Morphofunctional Considerations

#### *UCN3* mRNA Expression and Synthesis

Here we present the first demonstration of the distribution of *UCN3* mRNA and UCN3-ir labeled cells and fibers in the *Sapajus* spp. monkey brain. The major sites of *UCN3* mRNA expression in the monkey CNS are the Pa and the MnPO, with *UCN3* mRNA also found in the neurons of the SO and BM; the medial part of the CeM, Me, and AA; and the STMA and STMV. The prevalence of *UCN3* mRNA in the hypothalamic and amygdaloid areas contrasts to what occurs with other members of the CRF peptidergic family, especially CRF (Lewis et al., 2001; Li et al., 2002; Venihaki et al., 2004). These results agree with previous studies performed in other species, corroborating the conservation of this neuropeptidergic system (Hsu and Hsueh, 2001; Lewis et al., 2001; Li et al., 2002).

It is noteworthy that UCN3-ir cells could not be found in the monkey MnPO, even though its coding mRNA is present in this region. This result reflects controversial data in the literature regarding UCN3 synthesis in the MnPO, since Lewis et al. (2001) report the MnPO as the rodent region containing the highest number of mRNA-expressing and immunoreactive cells, while Li et al. (2002) described the presence of only a small group of UCN3-ir cells in this area. When taken together, these data suggest that complex post-translational mechanisms regulate the synthesis of ppUCN3 from its coding mRNA. Furthermore, the MnPO appears to be especially sensitive to these mechanisms as the presence of *UCN3* mRNA does not correlate to neuronal immunoreactivity in this area of the monkey. Zmijewski and Slominski (2010) suggest that alternative splicing and post-translational mechanisms play a major role in CRF communication, and these mechanisms may have been conserved in other members of the CRF family; however, little is currently known about the specific modifications related to UCN3 production.

In the monkey hypothalamus, two regions concentrate UCN3-ir neurons, the JPLH and the Pa. Like the mRNA distribution, UCN3 synthesis is similar to what is observed in other species, once again indicating that the peptidergic system is well conserved (Hsu and Hsueh, 2001; Lewis et al., 2001; Li et al., 2002; Table 4). The first group, the JPLH, is a region located between the fornix and Pa which corresponds in rodents to the rostral PFA. Kuperman et al. (2010), using targeted overexpression of UCN3 in the PFA, showed that these neurons regulate anxiety-related behaviors, energy expenditure and glucose metabolism by projecting to the LS and the VMH. Therefore, they suggest that UCN3 and its receptor, CRF_2_, constitute the main component of brain responses to physiological and psychological changes. A similar synthesis of UCN3 by neurons in the JPLH could indicate that UCN3 has conserved its role in the three behavioral responses to stress in primates.

**Table 4:** Comparison between the distribution of UCN3-ir fibers and *CRF*_2_ mRNA in selective regions of monkeys and rat brains.

Region	*Sapajus/Rhesus* UCN3-ir Fibers/*CRF*_2_ mRNA^a^	*Rattus* UCN3-ir Fibers^b^/*CRF*_2_ mRNA^c^
Forebrain		
Amygdala		
Central nucleus	++/+	−/−
Medial nucleus	+/−|+	+++/++
Septum		
Lateral nucleus	+++/++	++++/++++
Bed nucleus, stria terminalis		
Rostral region	+/++	+/−
Posterior region	+/++	+++/++
Hypothalamus		
Arcuate nucleus	++/NA	++/+
Medial preoptic nucleus	++/NA	+++/+|++
Paraventricular nucleus		
Parvicellular part	+/+++	+/+
Magnocellular part	+++/+++	+/+
Supraoptic nucleus	+/+++	−|+/++
Ventromedial nucleus	+++/+++	++++/+++
Hindbrain		
Nucleus of tract solitary	+/−	−/++
Periaqueductal gray	+/NA	+|++/+

*The presence of UCN3-ir fibers [Sapajus and Rattus^b^] a CRF_2_ mRNA [Rhesus^a^ and Rattus^c^ are indicated by a plus sign, and their absence is indicated by a minus sign. The relative density of Sapajus UCN3-ir fibers in different brain regions agrees to the patterns shown in Figure 1, indicated by the number of plus signs depending on the amount present in a specific region or with a minus sign to indicate the absence of labeled fibers, and non-available data is indicated by NA. ^a^Adapted from Sánchez et al. (1999).^b^Adapted from Li et al. (2002). ^c^Van Pett et al. (2000)*.

There is evidence that UCN3 is a highly synergistic peptide. As described by Wittmann et al. (2009), almost all UCN3-ir cells in the PFA produce thyrotropin-releasing hormone (TRH), while approximately half of all BNST neurons are UCN3/TRH. They also report that double-labeled UCN3/TRH neurons densely innervate the VMH. In addition, injection of UCN3 into the VMH decreases food intake (Fekete et al., 2007; Li et al., 2007) similar to injections of TRH, reinforcing that PFA UCN3 cells may be involved in food intake and glucose metabolism regulation, most likely in association with the TRH peptidergic system through the VMH in rodents.

Fekete et al. (2007) also reported a similar observation concerning the intracerebroventricular (icv) administration of leptin, suggesting a possible functional relationship between leptin and CRF_2_ in the VMH to the modulation of homeostasis (Fekete et al., 2007; Kuperman and Chen, 2008). Since there the presence of UCN3-ir labeled cells in the monkey JPLH associated with UCN3-ir fibers in the ST and VMH, taken together, these data suggest an anatomical substrate for the same regulation in primates, although more studies are necessary to clarify this function in rodents and primates.

The second group, located at caudal levels of the PaMD with moderate UCN3-ir labeled cells, was also identified in the neighboring PaPD, by immunoperoxidase and immunofluorescence. As established by Chen et al. (2011), the main UCN3 innervation of the VMH arises from the PVH and the posterior BNST, while the more posterior PFA neurons contribute more to the innervation of the LS. Interestingly, UCN3 injections into the PVH also have an anorectic effect, once more indicating the participation of UCN3 in energy homeostasis regulation (Chen et al., 2011). Recently, the same group showed that there are reciprocal connections between VMH and anterior parvicellular part of the paraventricular nucleus of the hypothalamus (PVHap; van-Hover and Li, 2015). Thus, this could be related to the modulation of the stress-associated behavior. The abundance of UCN3-ir cells in the Pa of *Sapajus* suggests a similar anatomical basis for this regulation in monkeys. The former group also described that PVHap receives information from different areas, such as LS, bed nucleus of the stria terminalis (BST) and amygdala, these nuclei are implicated in the modulation of learning and behavior, concerning autonomic regulation. These findings confirmed that the expression of *UCN3* in the MeA is sensitive to stress (Jamieson et al., 2006), and that the restraint stress increases the *UCN3* expression in the PVHap and BST, but no alteration was found in the rostral perifornical hypothalamus (van-Hover and Li, 2015). Despite the phylogenetic differences between rodents and monkeys, it is possible that this mechanism is conserved in non-human primates.

Nevertheless, a possible role for UCN3 in the Pa is not restricted to the modulation of motivational behaviors. Li et al. (2010) reported the involvement of UCN3 on the cardiovascular activity in rats. Following icv injections of UCN3 into the PVH, they observed a significant increase in systolic blood pressure, heart rate and renal sympathetic activity, implying that UCN3 plays a role in the neural control of cardiovascular function, by increasing sympathetic outflow through CRF_2_ activation in the PVH. Consistent with this notion, and comparing the functional data from the literature to our anatomical data, we suggest that UCN3 could have the similar functions in monkeys that it has in rodents through the Pa.

#### UCN3-ir Projections in Monkey CNS

The distribution of UCN3-ir fibers is restricted to some diencephalic territories displaying a very dense innervation. Among all regions, the most prominent UCN3-ir terminal fields are found in the VMHDM and the LSI; this finding is in full agreement with the UCN3 innervation density observed in the rodent brain (Li et al., 2002).

The VMH, Arc, lateral hypothalamus and PVH nuclei are important regions related to the central regulation of feeding, glucose homeostasis and energy balance and, also express high levels of *CRF_2_* (Lovenberg et al., 1995b; Van Pett et al., 2000; Li et al., 2002; Chen et al., 2005; Kuperman and Chen, 2008). There is also a partial overlap between the distribution of UCN3-ir fibers in the *Sapajus* spp. brain and the CRF_2_ mRNA expression sites in both rodents and non-human primates (Sánchez et al., 1999; Van Pett et al., 2000; Li et al., 2002).

Most CRF_2_-expressing neurons in the VMH have a glutamatergic excitatory activity, and a subset of those cells project to the ARH, more specifically to POMC-producing neurons (Chen et al., 2010). The ARH nucleus is recognized as a key anorectic component of the CNS, but it also plays a major role in regulating blood glucose levels (Chen et al., 2010). Thus, because activation of VMH CRF_2_ positive neurons leads to the stimulation of POMC cells in the ARH, food and blood glucose homeostasis can be achieved through UCN3 modulation (Chen et al., 2010). This result was further confirmed by Chen et al. (2011) by performing icv injections of UCN3 into the VMH of rats, which led to increased blood glucose levels, suppressed food intake and increased expression of POMC mRNA in the ARH. However, there was no activation of the hypothalamic-pituitary-adrenal axis (HPA) when compared to the control group. These findings suggest that CRF_2_ activation could modulate these nuclei, and that UCN3 neurons in these nuclei could be involved in modulating energy balance and homeostasis.

In rats, Singewald et al. (2011) hypothesized that the LS exerts a modulatory role in behavioral and neuroendocrine responses to stress. These authors report that the rat LSI plays a vital role in promoting an inhibitory influence over the HPA unlike the negative feedback mechanism of glucocorticoids. It also plays a role in enabling response strategies during exposure to stress. Thus, they support the hypothesis that LS neuronal activity is needed to cope with stress responses and provide protection against the deleterious effects of excessive exposure to psychological stress (Singewald et al., 2011).

Regarding the regions of mismatch, Sánchez et al. (1999) described that the choroid plexus region has the highest signal following *in situ* hybridization for the CRF_2_ mRNA in *Rhesus* monkeys. Although some UCN3 fibers are found near ependymal cells around the third ventricle, this innervation density is lower compared to the CRF_2_ mRNA expression in the choroid plexus. This finding suggests that UCN3 is not the main peptide that binds CRF_2_ in the choroid plexus. In the hypophysis, a similar mismatch occurs because UCN3-ir fibers are found in the *Sapajus* posterior lobe, while *CRF*_2_ mRNA is found exclusively in the anterior lobe (AL) of the *Rhesus* monkey (Sánchez et al., 1999). Interestingly, this hypophysial distribution of UCN3-ir fibers in *Sapajus* spp. agrees with the *CRF*_2_ mRNA distribution in rats found by Van Pett et al. (2000), which could reflect a possible divergence in the neuroendocrine mechanisms for the UCN3/CRF_2_ system between New and Old World monkeys (Table 4).

This partial overlap between UCN3-ir fibers and *CRF_2_* mRNA expression in primates has been similarly reported in rodents by Li et al. (2002). Conversely, Vasconcelos et al. (2003) also described limited matching regions between UCN1-ir fibers in the monkey CNS and the rodent CRF_2_ mRNA distribution (Chalmers et al., 1995; Van Pett et al., 2000). Nevertheless, the areas with the densest innervation of UCN3-ir fibers correlate with those containing CRF_2_ in other animal models (Lewis et al., 2001; Li et al., 2002; Jamieson et al., 2006; Kuperman et al., 2010), which therefore, suggests that UCN3 has been conserved as one of the endogenous ligands for CRF_2_ in non-human primates.

Since UCN3 is the only member of the CRF family which is a highly selective ligand for CRF_2_ (Lovenberg et al., 1995a; Hsu and Hsueh, 2001; Lewis et al., 2001; Deussing et al., 2010), we believe that the UCN3-ir fiber distribution provides an anatomical basis for UCN3 modulation of physiological effects related to endocrine and behavioral responses to stress in primates similar to rodents (Reul and Holsboer, 2002; Venihaki et al., 2004; Jamieson et al., 2006; Chen et al., 2010, 2012).

Venihaki et al. (2004) reported that UCN3 participated in the modulation of stress by alleviating some anxiety-related behaviors, despite UCN3 not affecting the activity of the HPA axis. The anxiolytic effects of UCN3 may serve two purposes. First, UCN3 may limit the extent of behavioral response to stress and thus may be beneficial for preventing excessive anxiety, which, in turn, may be an appropriate response to a stressful situation. Second, this limitation of the stress response may prevent the emergence of pathological responses to stress, which are one of the leading causes of psychiatric diseases, such as chronic anxiety disorders and depression in humans (Venihaki et al., 2004). The data presented here for *Sapajus* spp. further strengthen this notion, indicating that the UCN3 peptidergic system is optimally located to mediate and modulate stress responses in non-human primates.

## Conclusion

The main sites of UCN3-producing neurons in *Sapajus* spp. are the paraventricular nucleus of the hypothalamus, the PFA and the juxtaparaventricular lateral nucleus, which are similar to those described for rodents. The presence of UCN3-ir fibers mainly in the ventromedial nucleus of the hypothalamus, paraventricular nucleus of the hypothalamus and in the LSI is coincidental to the CRF_2_ distribution in the *Rhesus* monkey. When taken together, these data suggest that UCN3 plays a role in stress responses and neuroendocrinological control.

## Author Contributions

In this study, all authors had full access to the data and take responsibility for the data integrity and the accuracy of the data analysis. JCB: study concepts and design; study supervision. DSB: acquisition of the data. DSB and JCB: analysis and interpretation of the data; obtained funding. DSB, JCB, GBD: drafting of the manuscript. JCB, CFPL, DAL, LVS: critical revision of the manuscript for important intellectual content. PLC, JAO, CAC, KRTS, JMS, ARO: administrative, technical and material support.

## Conflict of Interest Statement

The authors declare that the research was conducted in the absence of any commercial or financial relationships that could be construed as a potential conflict of interest.

## Abbreviations

3V: 3rd ventricle
aa: amino acids
ac: anterior commissure
acp: anterior commissure, posterior limb
AA: anterior amygdaloid area
AH: anterior hypothalamic area
AHiPC: amygdalohippocampal area, parvicellular part
AL: anterior lobe of the hypophysis
AMPO: anteromedial preoptic nucleus
Arc: arcuate hypothalamic nucleus
ARH: rat arcuate nucleus
BM: basomedial amygdaloid nucleus
BMMC: basomedial amygdaloid nucleus, magnocellular part
BST: rat bed nucleus of the stria terminalis
BSTp: rat posterior part of BST
CA4: field CA4 of the hippocampus
Cd: caudate nucleus
Ce: central nucleus of the amygdala
CeI: dorsal motor nucleus of vagus, centrointermediate part
CeM: central amygdaloid nucleus, medial division
Co: cortical nucleus of the amygdala
CM: central medial thalamic nucleus
CL: centrolateral thalamic nucleus
CRF: corticotropin-releasing factor
CRF_1_: corticotropin-releasing factor receptor, subtype 1
CRF_2_: corticotropin-releasing factor receptor, subtype 2
DAB: 3,3′-Diaminobenzidine
f: fornix
GrDG: granule cell layer of the dentate gyrus
hnt: hypothalamo-neurohypophysial tract
HFD: *high-fat diet*
ic: internal capsule
icv: intracerebroventricular
ip: intraperitoneal injection
JPLH: juxtaparaventricular part of lateral hypothalamus
*ko/UCN3*: “*knockout”* UCN3
LH: lateral hypothalamic area
LHb: lateral habenular nucleus
LPAG: lateral periaqueductal gray
LPO: lateral preoptic nucleus
LS: lateral septal nucleus
LSI: LS, intermediate part
LSV: LS, ventral part
LTN: lateral tuberal nucleus
LV: lateral ventricle
Me: medial amygdaloid nucleus
MeA: rat medial amygdaloid nucleus
ME: median eminence
MePO: rat median preoptic nucleus
MHb: medial habenular nucleus
MnPO: median preoptic nucleus
MPA: medial preoptic area
MPO: medial preoptic nucleus
MS: medial septal nucleus
NAS: nickel ammonium sulfate
Sol: solitary nucleus
opt: optic tract
Pa: monkey paraventricular nucleus of the hypothalamus
PaMD: paraventricular hypothalamic nucleus, magnocellular part, dorsal division
PaPD: paraventricular hypothalamic nucleus, parvicellular part, dorsal division
PaPV: paraventricular hypothalamic nucleus, parvicellular part, ventral division
PC: paracentral thalamic nucleus
Pe: periventricular hypothalamic nucleus
PeF: perifornical nucleus
PH: rat posterior hypothalamic area
PI: *pars intermedia* of the hypophysis
PL: posterior lobe of the hypophysis
ppUCN3: prepro-UCN3
PVH: rat paraventricular nucleus of the hypothalamus
PVHap: anterior parvicellular part of the paraventricular nucleus of the hypothalamus
rCRF: rat CRF
rUCN3: rat UCN3
SCP: stresscopin
SFi: septofimbrial nucleus
SO: supraoptic nucleus
sox: supraoptic decussation
Sp5C: spinal trigeminal nucleus, caudal part
ST: bed nucleus of stria terminalis
STLD: ST, lateral division, dorsal part
STMA: ST, medial division, anterior part
STMV: ST, medial division, ventral part
TRH: thyrotropin-releasing hormone
TSH: thyroid stimulating hormone
UCN1: Urocortin 1
UCN2: Urocortin 2
UCN3: Urocortin 3
UCN3-ir: Urocortin 3 immunoreactivity
VGLUT2: second conveyor vesicular glutamate
VLPAG: periaqueductal gray matter, ventrolateral part
VMH: ventromedial hypothalamic nucleus
VMHDM: dorsomedial part of VMH
VMHVL: ventrolateral part of VMH
*wt/UCN3*: wild-type UCN3.

## References

[B1] Alfaro J. W. L. Silva J. D. S. E. Rylands A. B. (2012). How different are robust and gracile capuchin monkeys? An argument for the use of Sapajus and Cebus. Am. J. Primatol. 74, 273–286. 10.1002/ajp.22007 22328205

[B2] Bittencourt J. C. Vaughan J. Arias C. Rissman R. A. Vale W. W. Sawchenko P. E. (1999). Urocortin expression in rat brain: evidence against a pervasive relationship of urocortin-containing projections with targets bearing type 2 CRF receptors. J. Comp. Neurol. 415, 285–312. 10.1002/(sici)1096-9861(19991220)415:3<285::AID-CNE1>3.0.CO;2-0 10553117

[B3] Blin N. Stafford D. W. (1976). A general method for isolation of high molecular weight DNA from eukaryotes. Nucleic Acids Res. 3, 2303–2308. 10.1093/nar/3.9.2303 987581 PMC343085

[B4] Chalmers D. T. Lovenberg T. W. De Souza E. B. (1995). Localization of novel corticotropin-releasing factor receptor (CRF2) mRNA expression to specific subcortical nuclei in rat brain: comparison with CRF1 receptor mRNA expression. J. Neurosci. 15, 6340–6350. 7472399 10.1523/JNEUROSCI.15-10-06340.1995 PMC6577987

[B6] Chen P. Hover C. V. Lindberg D. Li C. (2012). Central urocortin 3 and type 2 corticotropin-releasing factor receptor in the regulation of energy homeostasis: critical involvement of the ventromedial hypothalamus. Front. Endocrinol. 3:180. 10.3389/fendo.2012.00180 23316185 PMC3539675

[B7] Chen P. Lin D. Giesler J. Li C. (2011). Identification of urocortin 3 afferent projection to the ventromedial nucleus of the hypothalamus in rat brain. J. Comp. Neurol. 519, 2023–2042. 10.1002/cne.22620 21452217 PMC3694597

[B5] Chen A. M. Perrin M. H. Digruccio M. R. Vaughan J. M. Brar B. K. Arias C. M.. (2005). A soluble mouse brain splice variant of type 2α corticotropin-releasing factor (CRF) receptor binds ligands and modulates their activity. Proc. Natl. Acad. Sci. U S A 102, 2620–2625. 10.1073/pnas.0409583102 15701705 PMC549000

[B8] Chen P. Vaughan J. Donaldson C. Vale W. Li C. (2010). Injection of Urocortin 3 into the ventromedial hypothalamus modulates feeding, blood glucose levels, and hypothalamic POMC gene expression but not the HPA axis. Am. J. Physiol. Endocrinol. Metab. 298, E337–E345. 10.1152/ajpendo.00402.2009 19952342 PMC2822480

[B9] Cox K. H. Deleon D. V. Angerer L. M. Angerer R. C. (1984). Detection of mRNAs in sea urchin embryos by *in situ* hybridization using asymmetric RNA probes. Dev. Biol. 101, 485–502. 10.1016/0012-1606(84)90162-3 6692991

[B10] Deussing J. M. Breu J. Kühne C. Kallnik M. Bunck M. Glasl L.. (2010). Urocortin 3 modulates social discrimination abilities via corticotropin-releasing hormone receptor type 2. J. Neurosci. 30, 9103–9116. 10.1523/jneurosci.1049-10.2010 20610744 PMC6632482

[B11] Fekete E. M. Inoue K. Zhao Y. Rivier J. E. Vale W. W. Szücs A.. (2007). Delayed satiety-like actions and altered feeding microstructure by a selective type 2 corticotropin-releasing factor agonist in rats: intra-hypothalamic urocortin 3 administration reduces food intake by prolonging the post-meal interval. Neuropsychopharmacology 32, 1052–1068. 10.1038/sj.npp.1301214 17019404 PMC2748839

[B12] Fragaszy D. Visalberghi E. Fedigan L. (2004). The Complete Capuchin: The Biology of the Genus Cebus. Cambridge: Cambridge University Press.

[B13] Gattass R. Sousa A. P. Rosa M. G. (1987). Visual topography of V1 in the Cebus monkey. J. Comp. Neurol. 259, 529–548. 10.1002/cne.902590404 3597827

[B14] Hauger R. L. Grigoriadis D. E. Dallman M. F. Plotsky P. M. Vale W. W. Dautzenberg F. M. (2003). International Union of Pharmacology. XXXVI. Current status of the nomenclature for receptors for corticotropin-releasing factor and their ligands. Pharmacol. Rev. 55, 21–26. 10.1124/pr.55.1.3 12615952

[B15] Hoffman G. E. Le W. W. Sita L. V. (2008). The importance of titrating antibodies for immunocytochemical methods. Curr. Protoc. Neurosci. 45, 2.12.1–2.12.26. 10.1002/0471142301.ns0212s45 18972376

[B16] Hsu S. Y. Hsueh A. J. (2001). Human stresscopin and stresscopin-related peptide are selective ligands for the type 2 corticotropin-releasing hormone receptor. Nat. Med. 7, 605–611. 10.1038/87936 11329063

[B17] Izar P. Verderane M. P. Peternelli-Dos-Santos L. Mendonça-Furtado O. Presotto A. Tokuda M.. (2012). Flexible and conservative features of social systems in tufted capuchin monkeys: comparing the socioecology of Sapajus libidinosus and Sapajus nigritus. Am. J. Primatol. 74, 315–331. 10.1002/ajp.20968 21656840

[B18] Jamieson P. M. Li C. Kukura C. Vaughan J. Vale W. (2006). Urocortin 3 modulates the neuroendocrine stress response and is regulated in rat amygdala and hypothalamus by stress and glucocorticoids. Endocrinology 147, 4578–4588. 10.1210/en.2006-0545 16809443

[B19] Kang H. J. Adams D. H. Simen A. Simen B. B. Rajkowska G. Stockmeier C. A.. (2007). Gene expression profiling in postmortem prefrontal cortex of major depressive disorder. J. Neurosci. 27, 13329–13340. 10.1523/jneurosci.4083-07.2007 18045927 PMC3763487

[B20] Kuperman Y. Chen A. (2008). Urocortins: emerging metabolic and energy homeostasis perspectives. Trends Endocrinol. Metab. 19, 122–129. 10.1016/j.tem.2007.12.002 18337115

[B21] Kuperman Y. Issler O. Regev L. Musseri I. Navon I. Neufeld-Cohen A.. (2010). Perifornical Urocortin-3 mediates the link between stress-induced anxiety and energy homeostasis. Proc. Natl. Acad. Sci. U S A 107, 8393–8398. 10.1073/pnas.1003969107 20404164 PMC2889556

[B22] Lewis K. Li C. Perrin M. Blount A. Kunitake K. Donaldson C.. (2001). Identification of urocortin III, an additional member of the corticotropin-releasing factor (CRF) family with high affinity for the CRF2 receptor. Proc. Natl. Acad. Sci. U S A 98, 7570–7575. 10.1073/pnas.121165198 11416224 PMC34709

[B23] Li C. Chen P. Vaughan J. Lee K.-F. Vale W. (2007). Urocortin 3 regulates glucose-stimulated insulin secretion and energy homeostasis. Proc. Natl. Acad. Sci. U S A 104, 4206–4211. 10.1073/pnas.0611641104 17360501 PMC1820733

[B25] Li X. Fan M. Shen L. Cao Y. Zhu D. Hong Z. (2010). Excitatory responses of cardiovascular activities to urocortin3 administration into the PVN of the rat. Auton. Neurosci. 154, 108–111. 10.1016/j.autneu.2009.12.004 20060787

[B24] Li C. Vaughan J. Sawchenko P. E. Vale W. W. (2002). Urocortin III-immunoreactive projections in rat brain: partial overlap with sites of type 2 corticotrophin-releasing factor receptor expression. J. Neurosci. 22, 991–1001. 11826127 10.1523/JNEUROSCI.22-03-00991.2002 PMC6758528

[B26] Lovenberg T. W. Chalmers D. T. Liu C. De Souza E. B. (1995a). CRF2 α and CRF2 β receptor mRNAs are differentially distributed between the rat central nervous system and peripheral tissues. Endocrinology 136, 4139–4142. 10.1210/endo.136.9.7544278 7544278

[B27] Lovenberg T. W. Liaw C. W. Grigoriadis D. E. Clevenger W. Chalmers D. T. De Souza E. B.. (1995b). Cloning and characterization of a functionally distinct corticotropin-releasing factor receptor subtype from rat brain. Proc. Natl. Acad. Sci. U S A 92, 836–840. 10.1073/pnas.92.12.5759-b 7846062 PMC42715

[B28] Lynch Alfaro J. W. Boubli J. P. Olson L. E. Di Fiore A. Wilson B. Gutiérrez-Espeleta G. A. (2012). Explosive Pleistocene range expansion leads to widespread Amazonian sympatry between robust and gracile capuchin monkeys. J. Biogeogr. 39, 272–288. 10.1111/j.1365-2699.2011.02609.x

[B29] Manocha S. L. Shantha T. R. Bourne G. H. (1968). A Stereotaxic Atlas of the Brain of the Cebus Monkey (Cebus Apella). London: Oxford University Press.

[B30] Paxinos G. Huang X. F. Petrides M. Toga A. W. (2009). The Rhesus Monkey Brain—In Stereotaxic Coordinates. San Diego, CA: Elsevier.

[B31] Paxinos G. Watson C. Petrides M. Rosa M. Tokuno H. (2012). The Marmoset Brain in Stereotaxic Coordinates. London: Academic Press, Elsevier.

[B32] Pinato L. Frazão R. Cruz-Rizzolo R. Cavalcante J. Nogueira M. (2009). Immunocytochemical characterization of the pregeniculate nucleus and distribution of retinal and neuropeptide Y terminals in the suprachiasmatic nucleus of the Cebus monkey. J. Chem. Neuroanat. 37, 207–213. 10.1016/j.jchemneu.2009.01.005 19481005

[B33] Reul J. M. Holsboer F. (2002). Corticotropin-releasing factor receptors 1 and 2 in anxiety and depression. Curr. Opin. Pharmacol. 2, 23–33. 10.1016/s1471-4892(01)00117-5 11786305

[B34] Reyes T. M. Lewis K. Perrin M. H. Kunitake K. S. Vaughan J. Arias C. A.. (2001). Urocortin II: a member of the corticotropin-releasing factor (CRF) neuropeptide family that is selectively bound by type 2 CRF receptors. Proc. Natl. Acad. Sci. U S A 98, 2843–2848. 10.1073/pnas.051626398 11226328 PMC30227

[B35] Sánchez M. M. Young L. J. Plotsky P. M. Insel T. R. (1999). Autoradiographic and *in situ* hybridization localization of corticotropin-releasing factor 1 and 2 receptors in nonhuman primate brain. J. Comp. Neurol. 408, 365–377. 10.1002/(SICI)1096-9861(19990607)408:3<365::AID-CNE5>3.0.CO;2-N 10340512

[B60] Sawchenko P. E. Swanson L. W. Vale W. W. (1984). Corticotropin-releasing factor: co-expression within distinct subsets of oxytocin-, vasopressin-, and neurotensin-immunoreactive neurons in the hypothalamus of the male rat. J. Neurosci. 4, 1118–1129. 6609226 10.1523/JNEUROSCI.04-04-01118.1984 PMC6564788

[B36] Silva J. D. S. Jr. (2001). Especiação nos Macacos-Prego e Caiararas, Gênero Cebus Erxleben, 1777 (Primates, Cebidae). Ph.D. Dissertation, Programa de Pós-Graduação em Genética, Universidade Federal do Rio de Janeiro, Rio de Janeiro.

[B37] Simmons D. M. Arriza J. L. Swanson L. W. (1989). A complete protocol for *in situ* hybridization of messenger RNAs in brain and other tissues with radio-labeled single-stranded RNA probes. J. Histotechnol. 12, 169–181. 10.1179/014788889794651870

[B38] Singewald G. M. Rjabokon A. Singewald N. Ebner K. (2011). The modulatory role of the lateral septum on neuroendocrine and behavioral stress responses. Neuropsychopharmacology 36, 793–804. 10.1038/npp.2010.213 21160468 PMC3055728

[B39] Swanson L. W. Sawchenko P. E. Rivier J. Vale W. W. (1983). Organization of ovine corticotropin-releasing factor immunoreactive cells and fibers in the rat brain: an immunohistochemical study. Neuroendocrinology 36, 165–186. 10.1159/000123454 6601247

[B40] Vale W. Spiess J. Rivier C. Rivier J. (1981). Characterization of a 41-residue ovine hypothalamic peptide that stimulates secretion of corticotropin and β-endorphin. Science 213, 1394–1397. 10.1126/science.6267699 6267699

[B61] van der Meulen T. Donaldson C. J. Cáceres E. Hunter A. E. Cowing-Zitron C. Pound L. D.. (2015). Urocortin3 mediates somatostatin-dependent negative feedback control of insulin secretion. Nat. Med. 21, 769–776. 10.1038/nm.3872 26076035 PMC4496282

[B42] van-Hover C. Li C. (2015). Stress-activated afferent inputs into the anterior parvicellular part of the paraventricular nucleus of the hypothalamus: insights into urocortin 3 neuron activation. Brain Res. 1611, 29–43. 10.1016/j.brainres.2015.03.009 25779038 PMC4441854

[B41] Van Pett K. Viau V. Bittencourt J. C. Chan R. K. Li H. Y. Arias C.. (2000). Distribution of mRNAs encoding CRF receptors in brain and pituitary of rat and mouse. J. Comp. Neurol. 428, 191–212. 10.1002/1096-9861(20001211)428:2<191::AID-CNE1>3.0.CO;2-U 11064361

[B43] Vasconcelos L. A. Donaldson C. Sita L. V. Casatti C. A. Lotfi C. F. Wang L.. (2003). Urocortin in the central nervous system of a primate (Cebus apella): sequencing, immunohistochemical, and hybridization histochemical characterization. J. Comp. Neurol. 463, 157–175. 10.1002/cne.10742 12815753

[B44] Vaughan J. Donaldson C. Bittencourt J. Perrin M. H. Lewis K. Sutton S.. (1995). Urocortin, a mammalian neuropeptide related to fish urotensin I and to corticotropin-releasing factor. Nature 378, 287–292. 10.1038/378287a0 7477349

[B45] Venihaki M. Sakihara S. Subramanian S. Dikkes P. Weninger S. C. Liapakis G.. (2004). Urocortin III, a brain neuropeptide of the corticotropin-releasing hormone family: modulation by stress and attenuation of some anxiety-like behaviours. J. Neuroendocrinol. 16, 411–422. 10.1111/j.1365-2826.2004.01170.x 15117334

[B46] Wittmann G. Füzesi T. Liposits Z. Lechan R. M. Fekete C. (2009). sDistribution and axonal projections of neurons coexpressing thyrotropin-releasing hormone and urocortin 3 in the rat brain. J. Comp. Neurol. 517, 825–840. 10.1002/cne.22180 19844978 PMC2849936

[B47] Zmijewski M. A. Slominski A. T. (2010). Emerging role of alternative splicing of CRF1 receptor in CRF signaling. Acta Biochim. Pol. 57, 1–13. 20234885 PMC2883312

