# Supplementary figures and images for: Anatomical Organization of Urocortin 3-Synthesizing Neurons and Immunoreactive Terminals in the Central Nervous System of Non-Human Primates [Sapajus spp.]
Source: Front Neuroanat. 2017 Jul 24;11:57. doi: 10.3389/fnana.2017.00057 (PMC5522884; doi:10.3389/fnana.2017.00057)

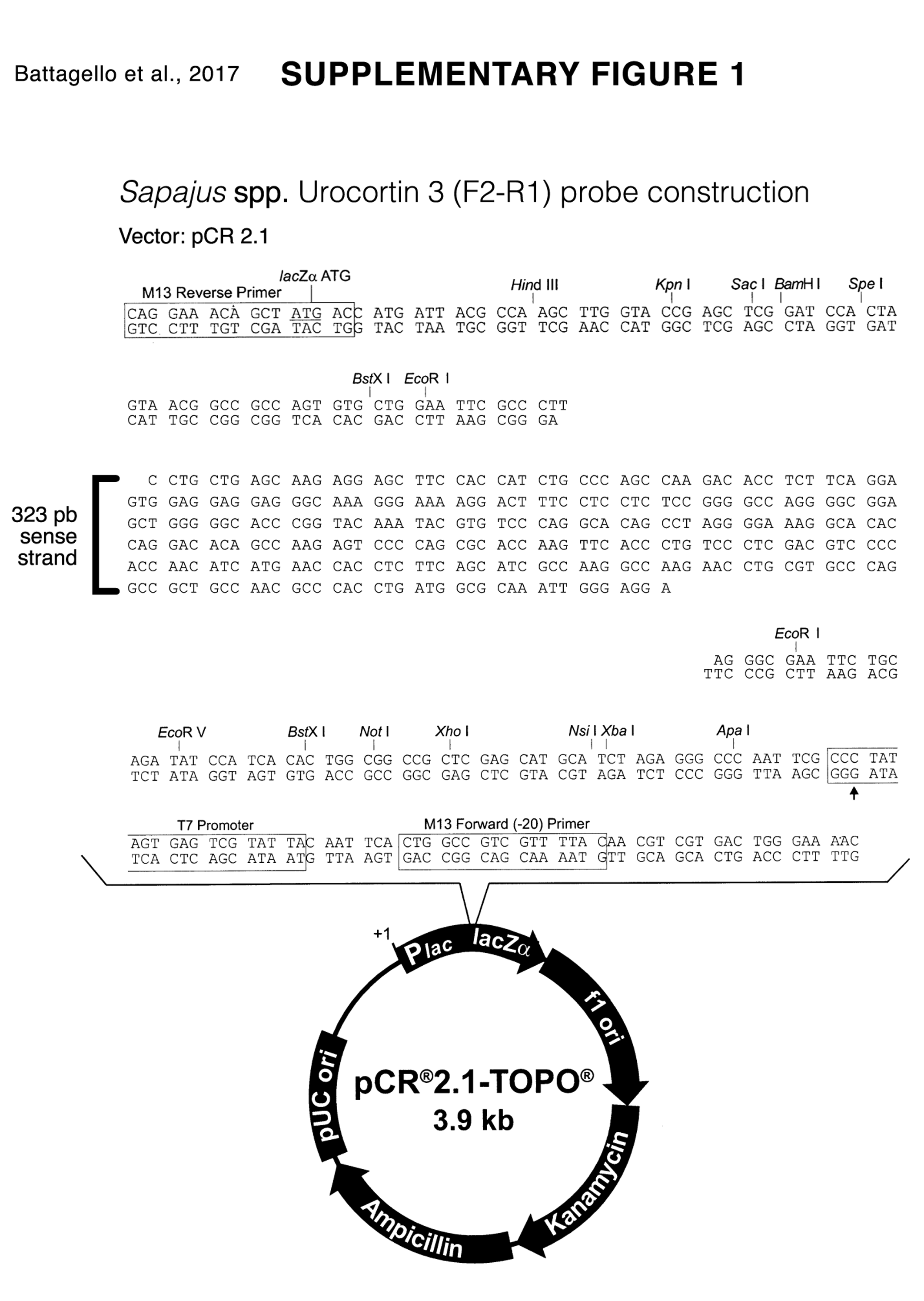

Supplement: Supplementary file 1 [file Image_1.tif]

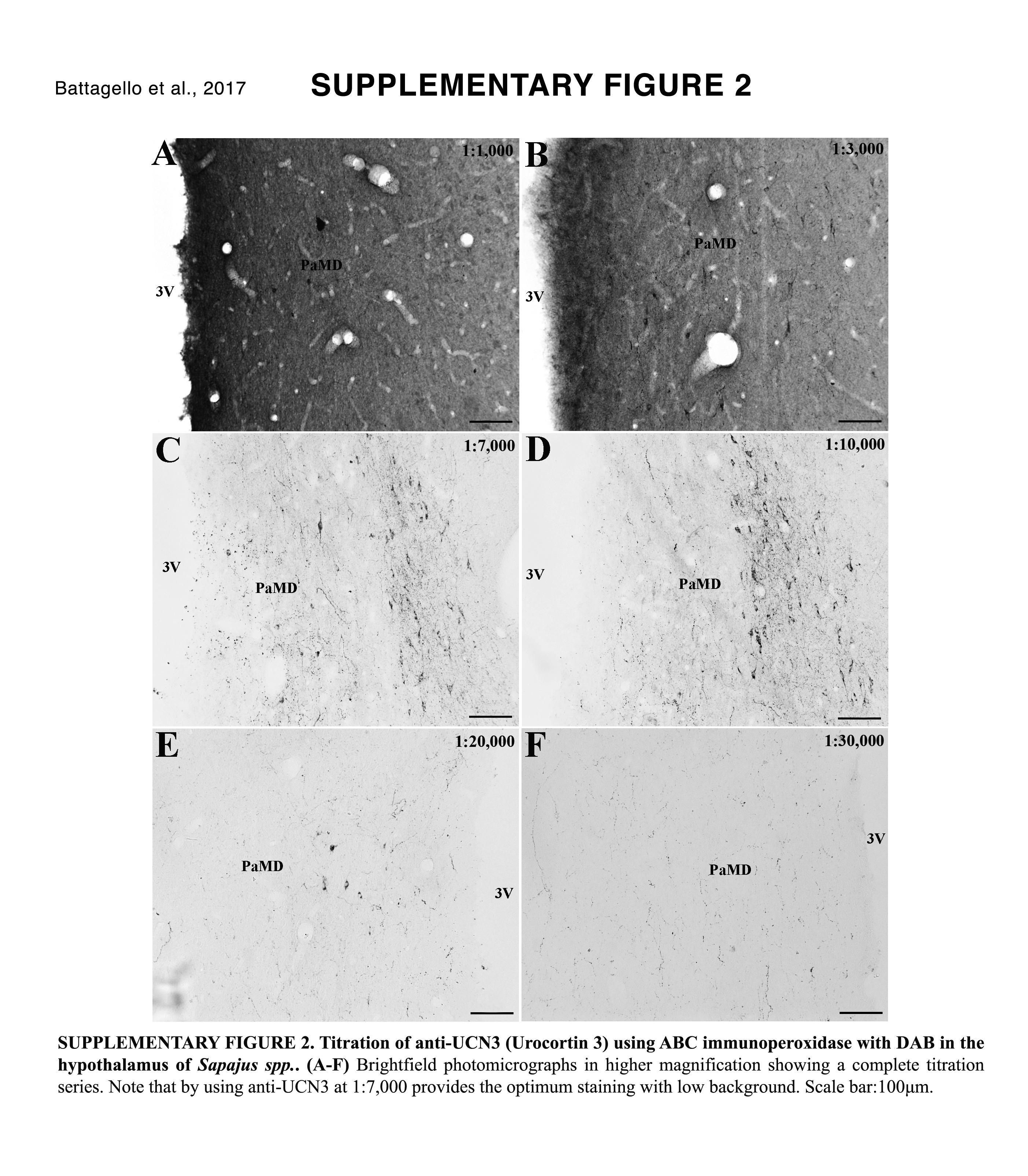

Supplement: Supplementary file 2 [file Image_2.tif]

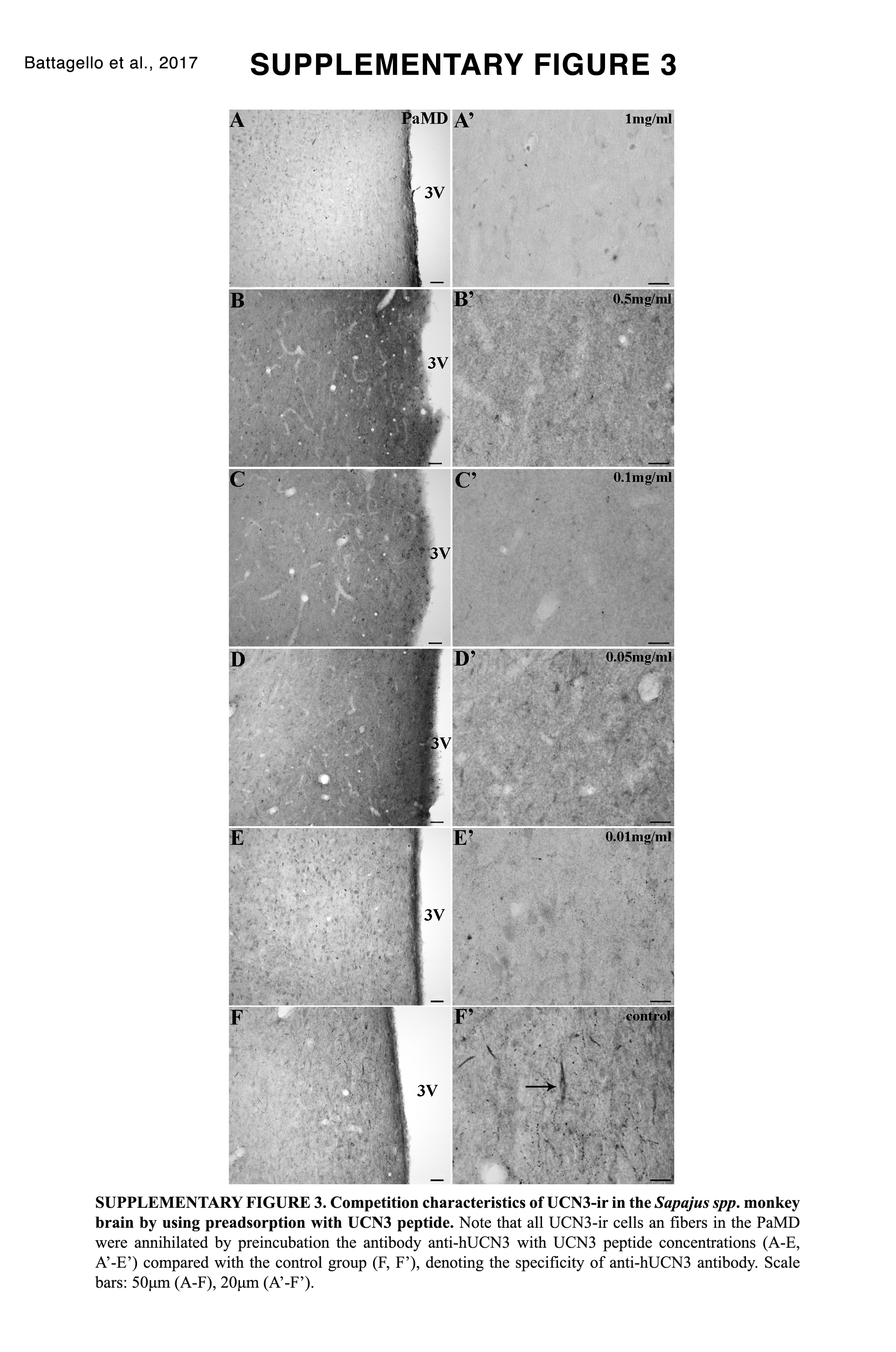

Supplement: Supplementary file 3 [file Image_3.tif]

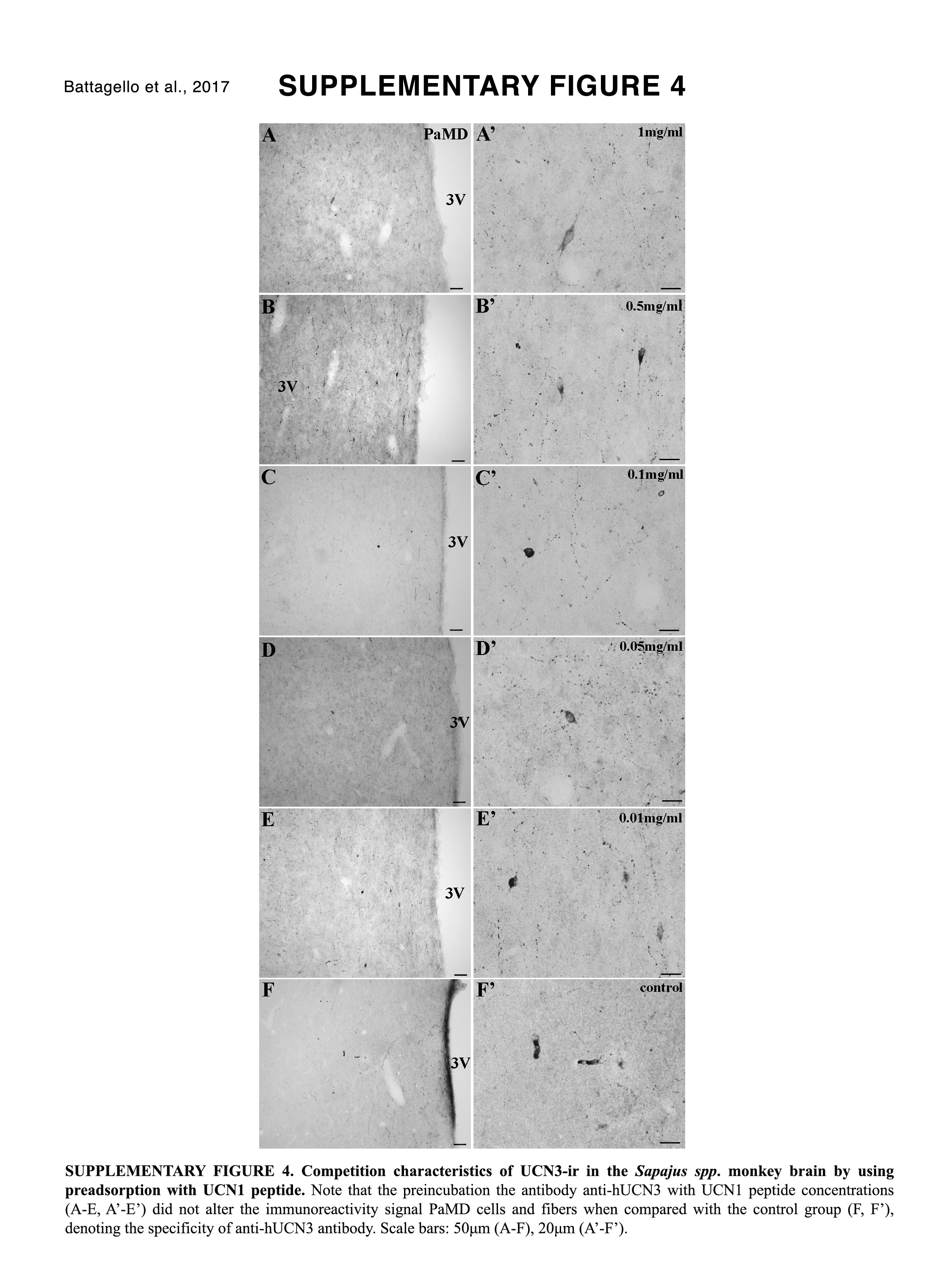

Supplement: Supplementary file 4 [file Image_4.tif]

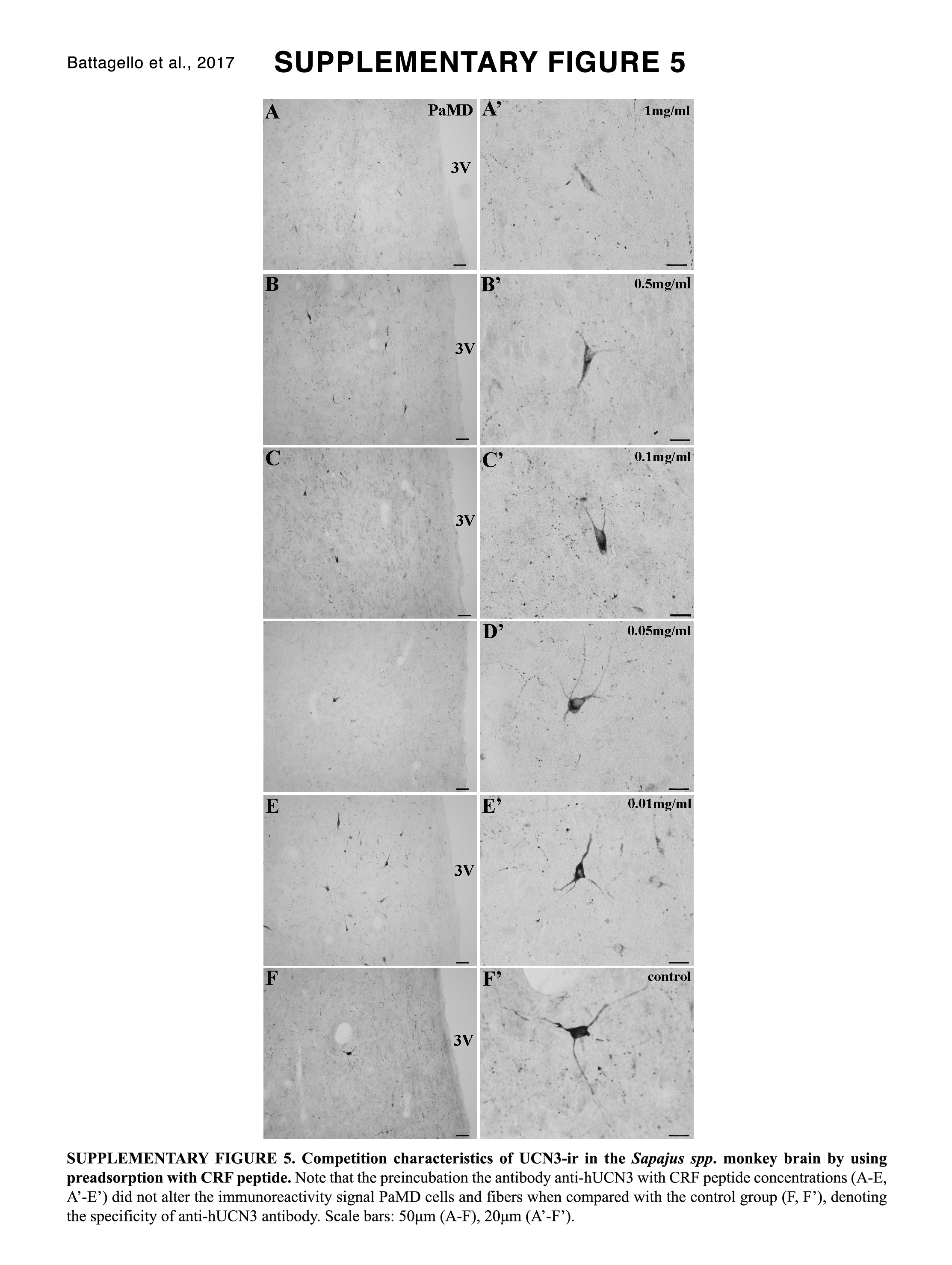

Supplement: Supplementary file 5 [file Image_5.tif]
